# Usefulness of a humanized tricellular static transwell blood–brain barrier model as a microphysiological system for drug development applications. - A case study based on the benchmark evaluations of blood-brain barrier microphysiological system

**DOI:** 10.1016/j.reth.2023.02.001

**Published:** 2023-02-24

**Authors:** Kimiko Nakayama-Kitamura, Yukari Shigemoto-Mogami, Hiroko Toyoda, Ikue Mihara, Hiroyuki Moriguchi, Hitoshi Naraoka, Tomomi Furihata, Seiichi Ishida, Kaoru Sato

**Affiliations:** a Laboratory of Neuropharmacology, Division of Pharmacology, National Institute of Health Science, 3-25-26 Tonomachi, Kawasaki-ku, Kawasaki City, Kanagawa, Japan; b Stem Cell Evaluation Technology Research Association, Grande Building 8F, 2-26-9 Hatchobori, Chuo-ku, Tokyo 104-0032, Japan; c School of Pharmacy, Tokyo University of Pharmacy and Life Sciences, 1432-1 Horinouchi, Hachioji, Tokyo 192-0392 Japan; d Division of Applied Life Science, Graduate School of Engineering, Sojo University, 4-22-1 Ikeda, Nishi-ku, Kumamoto City, Kumamoto, Japan

**Keywords:** Blood‒brain barrier (BBB), Microphysiological system (MPS), *P*-gp, BCRP, BBB, blood-brain barrier, CNS, central nervous system, HBMEC, Human brain microvascular endothelial cells, HASTR, Human astrocytes, HBPC, Human brain pericyte, *P*-gp, *P*-glycoprotein, BCRP, Breast cancer resistance protein, Glut1, Glucose transporter 1, TEER, *Trans*-endothelial electrical resistance, LY, Lucifer yellow, LC-MS/MS, Liquid chromatography with tandem mass spectrometry, MPS, Microphysiological system, TfR, Transferrin receptor

## Abstract

Microphysiological system (MPS), a new technology for in vitro testing platforms, have been acknowledged as a strong tool for drug development. In the central nervous system (CNS), the blood‒brain barrier (BBB) limits the permeation of circulating substances from the blood vessels to the brain, thereby protecting the CNS from circulating xenobiotic compounds. At the same time, the BBB hinders drug development by introducing challenges at various stages, such as pharmacokinetics/pharmacodynamics (PK/PD), safety assessment, and efficacy assessment. To solve these problems, efforts are being made to develop a BBB MPS, particularly of a humanized type. In this study, we suggested minimal essential benchmark items to establish the BBB-likeness of a BBB MPS; these criteria support end users in determining the appropriate range of applications for a candidate BBB MPS. Furthermore, we examined these benchmark items in a two-dimensional (2D) humanized tricellular static transwell BBB MPS, the most conventional design of BBB MPS with human cell lines. Among the benchmark items, the efflux ratios of *P*-gp and BCRP showed high reproducibility in two independent facilities, while the directional transports meditated through Glut1 or TfR were not confirmed. We have organized the protocols of the experiments described above as standard operating procedures (SOPs). We here provide the SOPs with the flow chart including entire procedure and how to apply each SOP. Our study is important developmental step of BBB MPS towards the social acceptance, which enable end users to check and compare the performance the BBB MPSs.

## 1 Introduction

In the central nervous system (CNS), the blood‒brain barrier (BBB) limits the permeation of circulating substances from the blood vessels to the brain parenchyma, thereby protecting the CNS from circulating xenobiotic compounds [1]. Essential nutrients (glucose, etc.) for the CNS are transported by specific transporters on vascular endothelial cells [2,3]. These BBB functions maintain the homeostatic CNS environment; however, they make drug development difficult by introducing challenges in the prediction of pharmacokinetics/pharmacodynamics (PKPD) [4], toxicokinetics/toxicodynamics (TKTD), toxicity, safety, and efficacy in the human CNS [5,6]. In addition, there are species differences in BBB functional proteins [7,8]. Humanized in vitro BBB models are now being studied with great interest as new tools to solve the issues described above [5].

Microphysiological system (MPS), a new technology for in vitro testing platforms, have been acknowledged as a strong tool for drug development. According to the draft definition proposed by the Food and Drug Administration (FDA), USA, an MPS is defined as “an in vitro platform composed of cells, explants derived from tissues/organs, and/or organoid cell formations of human or animal origin in a micro-environment that provides and supports biochemical/electrical/mechanical responses to model a set of specific properties that define organ or tissue function” [9]. According to this definition, in vitro BBB models developed so far are included in BBB MPS systems (BBB MPSs). We therefore launched a BBB MPS project in the industry-government-academia MPS initiative of Japan, an initiative that aims to establish MPSs in practical use for new drug development and to clarify the regulatory requirements. To date, a vast variety of BBB MPSs have been reported. For example, in terms of the device design, there are two-dimensional (2D) models, three-dimensional (3D) models, microfluidic models, etc. [10], and various combinations of cells are used in these systems. The challenge of developing humanized models is also being pursued. At present, end users must choose the optimal model for their study purpose based on their own criteria.

In this study, we therefore suggested a set of minimal essential benchmark items to establish the BBB-likeness of a BBB MPS; with these criteria, end users could determine the appropriate range of applications for a candidate BBB MPS. Furthermore, we examined these benchmark items in a 2D humanized tricellular static transwell BBB MPS [11], the most conventional type of BBB MPS with human cell lines (human immortalized brain microvascular endothelial cells: HBMEC/ci18; pericytes: HBVPC/ci37; astrocytes: HASTR/ci35) [[11], [12], [13], [14]]. We also validated the reproducibility of the protocols for a transporter activity assay with an independent facility and suggested an appropriate assay for this BBB MPS. Based on these experiments, we organized the standard operating procedures (SOPs) for evaluating the benchmark items in a 2D humanized tricellular static transwell BBB MPS [11] with the flow chart including entire procedure and how to apply each SOP.

## 2 Materials and methods

### 2.1 Materials

HBMEC/ci18, HBVPC/ci37, and HASTR/ci35 were established and supplied by Prof. Furihata. VascuLife complete medium was purchased from Kurabo (Osaka, Japan). Astrocyte growth medium, Neurobasal medium, fibronectin, *anti*-TfR antibody (#13–6800), and rhodamine 123 were purchased from Thermo Fisher Scientific (Waltham, USA). Pericyte medium was purchased from ScienCell Research Laboratories (Carlsbad, CA, USA). Blasticidin S was purchased from Fujifilm Wako (Tokyo, Japan). Collagen IV and collagen I were purchased from Nitta Gelatin (Osaka, Japan). *Anti*-Claudin-5 (ab131259), *anti*-*P*-gp (ab170904), and anti-Glut1 (ab115730) antibodies were purchased from Abcam (Cambridge, UK). *Anti*-β-actin antibody was purchased from Sigma–Aldrich (A5316, St. Louis, MO, USA). Anti-CD31 antibody was purchased from Proteintech (66065-1-Ig, Rosemont, IL, USA). *Anti*-ZO-1 antibody was purchased from Invitrogen (#339100). *Anti*-BCRP antibody was purchased from Cell Signaling Technology (#4477, Danvers, MA, USA). Anti-rabbit IgG conjugated with Alexa Fluor 488 or 594, anti-goat IgG conjugated with Alexa Fluor 488, and anti-mouse IgG conjugated with Alexa Fluor 488 or 594 were purchased from Molecular Probes. Fetal bovine serum (FBS) and Dulbecco's modified Eagle's medium (DMEM) were purchased from Life Technologies (Grand Island, NY, USA). Can Get Signal was purchased from TOYOBO (Osaka, Japan). Hoechst 33,342 and DAPI were purchased from Dojindo (Tokyo, Japan). 2-NBDG was purchased from Cayman Chemical Company (Ann Arbor, Michigan, USA). Digoxin was purchased from Alfer Aeser (Heysham, Lancashire, UK). Dantrolene and salazosulfapyridine (sulfasalazine, SASP) were purchased from Tocris Bioscience (Minneapolis, MN, USA). Adenosine 3′,5′-cyclic monophosphate sodium salt monohydrate was purchased from Merck (Darmstadt, Germany). Both human transferrin with no conjugated fluorophore and human transferrin conjugated with Alexa Fluor 488 were purchased from Jackson ImmunoResearch (West Grove, USA).

### 2.2 Cell cultures

Human brain microvascular endothelial cells/conditionally immortalized clone 18 (HBMEC/ci18), human brain pericytes/conditionally immortalized clone 37 (HBPC/ci37), and human astrocytes/conditionally immortalized clone 35 (HASTR/ci35) were established by Prof. Furihata et al. [[11], [12], [13], [14], [15]]. For maintenance, HBMEC/ci18 cultures were grown in VascuLife complete medium, and HASTR/ci35 and HBPC/ci37 cultures were grown in astrocyte growth medium and pericyte medium, respectively. All culture media contained 4 μg/ml blasticidin S. These cells were cultured at 33 °C for growth and at 37 °C for differentiation. Our specific experimental procedure has been standardized as the standard operating procedure (SOP) (Supplemental information).

### 2.3 Preparation of in vitro human BBB models

In vitro human BBB models were developed by combining three immortalized cell lines [11]). Briefly, HBPC/ci37 cells were seeded on the bottom side of the collagen IV- and Fibronectin-coated polycarbonate membrane of a transwell insert (Millicell cell culture insert 24-well hanging inserts, 0.4 μm PET; Merck, Darmstadt, Germany) at a density of 1.0 × 10^4^ cells/insert. The cells were then cultured for 1 day to allow them to attach firmly. HASTR/ci35 cells were seeded (5.0 × 10^4^ cells/well) on collagen I-coated 24-well plates (Greiner Bio-one, Frickenhausen, Germany) and maintained in astrocyte culture medium. HBPC/ci37 cells were induced to differentiate by replacing the pericyte medium with pericyte differentiation medium, which was consisted of FBS- and blasticidin *S*-free pericyte medium; HASTR/ci35 cells were induced to differentiate by replacing the astrocyte culture medium with astrocyte differentiation medium, which was consisted of FBS- and blasticidin *S*-free astrocyte growth medium supplemented with 1 mM adenosine 3′,5′-cyclic monophosphate sodium salt monohydrate. After the differentiation media were added, both cell lines were cultured at 37 °C for 24 h. To start a coculture, HBMEC/ci18 cells were seeded on the inner side of the HBPC/ci37 cell culture insert at a density of 1.0 × 10^5^ cells. Finally, the transwell inserts with HBMEC/ci18 cells and HBPC/ci37 cells were transferred into 24-well plates containing HASTR/ci35 cells. The cells were re-fed with VEGF- and EGF-free VascuLife complete medium in the inner insert and the Neurobasal medium with N2 supplement in the lower chamber. Day 0 was defined as the day of EC plating on the membrane. The cells were incubated at 33 °C.

On Day 1, the *trans*-endothelial electrical resistance (TEER) was measured by an EVOM^2^ voltohmmeter (World Precision Instruments, Sarasota, California, USA) with chopstick electrodes. The net resistance value was calculated by subtracting the measured resistance value of the insert membrane from the measured resistance value of the coculture. TEER (Ω × cm^2^) = the net resistance value (Ω) × surface area (cm^2^).

### 2.4 Total RNA isolation and quantitative reverse transcription real-time polymerase chain reaction (qRT‒PCR)

Total RNA extraction from cells in each model was conducted using an RNeasy Mini kit (Qiagen, Maryland, USA). RT‒PCR was performed with an ABI Prism 7300 sequence detection system (Applied Biosystems) using TaqPath1-step Multiplex Master Mix (Applied Biosystems) to determine the mRNA expression levels of the following: ZO-1, Claudin-5, *P*-gp, Glut1, BCRP, and transferrin receptor. The primer/probe sequences used were Hs01551861_m1, Hs00533949_s1, Hs00184500_m, Hs00892681_m1, and Hs01053790_m1 [[16], [17], [18], [19]]. Data were analyzed using a standard curve based on serial dilutions of total RNA from 3 types of cells.

### 2.5 Western blotting

The expression levels of TJ proteins and transporter proteins were analyzed by Western blotting [20]. After the examination of TEER, Western blots were performed to analyze protein extracts from both endothelial cells and pericytes. These extracts were obtained by lysing the cells with sample buffer (62.5 mM Tris, 2% SDS, 10% glycerin, 0.0125% bromophenol blue, pH 6.8) and homogenizing them on ice. The lysates were resolved by SDS‒PAGE and transferred to PVDF membranes. The membranes were incubated overnight in BlockAce blocking solution at 4 °C. Then, the membranes were incubated with primary antibodies for 1 h at 25 °C. After being washed three times, the membranes were incubated with horseradish peroxidase-conjugated anti-rabbit IgG or anti-mouse IgG antibody (1:5000) for 1 h at 25 °C. The membranes were then washed three times, and signals were visualized with a LAS3000 chemiluminescence detector (Fujifilm Co., Tokyo, Japan). We confirmed that the bands matched the molecular weights of the specific proteins of interest, i.e., CD31 (120 kDa), ZO-1 (225 kDa), Claudin-5Claudin-5 (24 kDa), *P*-gp (180 kDa), BCRP (65–80 kDa), Glut1 (40–60 kDa), TfR (90 kDa), and b-actin (42 kDa).

### 2.6 Immunostaining

The distribution of TJ proteins and transporter proteins was analyzed by immunocytochemistry [20]. After the examination of BBB functional activity, the cells from the blood side of the barrier (HBMEC/ciβ and HBPC/ci37) were fixed with 4% paraformaldehyde before 2 h of incubation in a blocking solution (3% normal goat serum, 0.3% Triton-X in PBS) at room temperature. Then, the cells were incubated in the primary antibody solution for 16 h at 4 °C. The concentrations of primary antibodies against the human proteins were as follows: anti-CD31 (×200), *anti*-ZO-1 (×100), *anti*-Claudin-5 (×100), *anti*-*P*-gp (×100), anti-Glut1 (×100), *anti*-BCRP (×100), and *anti*-TfR (×200). The cells were washed in 0.1% Triton-X PBS and then incubated in 0.1% Triton-X PBS containing secondary antibodies (anti-rabbit IgG conjugated with Alexa Fluor 488 or 594, anti-goat IgG conjugated with Alexa Fluor 488, anti-mouse IgG conjugated with Alexa Fluor 488 or 594, ×1000) for 3 h at room temperature. The cells were washed and counterstained with DAPI (×1000). The membranes were cut out and sandwiched between cover slips with an anti-fade protective agent. Fluorescent images were obtained using a Nikon A1R-A1 confocal microscope system (Nikon, Tokyo, Japan).

### 2.7 Permeability and bidirectional transport assays

Assays were performed in DPBS-H (10 mM HEPES, 25 mM glucose, in Dulbecco's phosphate-buffered saline with calcium chloride and magnesium chloride) in 24-well transwell plates on a rocking shaker at 20 rpm, 37 °C, 95% humidity, and 5% CO_2_. All substrates were dissolved at specific concentrations in DPBS-H and added to the luminal (A) or abluminal (B) side. The incubation times for the substrate were 15, 30, 45, and 60 min. The concentrations of the substrates were measured by a fluorescence microplate reader (Fluoroskan Ascent FL, Thermo Fisher Scientific). Digoxin, dantrolene, and salazosulfapyridine samples were pretreated with acetonitrile precipitation of proteins and measured using an LC-MS/MS system (ExionLC-QTRAP6500+, SCIEX, Framingham, Massachusetts, USA). The compound concentrations were as follows: rhodamine 123 (10 μM), Hoechst 33,342 (200 μM), 2-NBDG (100 μg/ml), digoxin (5 μM), dantrolene (5 μM), and salazosulfapyridine (sulfasalazine, SASP) (5 μM).

In this study, we employed P_e_ as the permeability coefficient because researchers can eliminate the influence of the insert membranes. The permeability coefficient (P_e_) was calculated according to Nakagawa et al. [16] by dividing the amount of substrate in the luminal compartment (A) by the substrate concentration in the abluminal compartment (B). The volume was obtained at multiple timepoints according to the following formula:

Volume (μL)=[C]r[R]/[C]d

where [C]r is the amount of the compound in the receiving compartment, [C]d represents the amount of the compound in the donor compartment, and [R] is the volume of the receiving compartment. When the volume is plotted over time, the slope equals the permeability × surface area product (PS) of the membrane. The PS of the membrane with cells is called the total PS (PS_total_), and the PS of the membrane without cells is called the membrane PS (PS_mem_). The P_e_ can be computed from the PS_total_ and PS_mem_:

1/PS_e_ = 1/PS_total_-1/PS_mem_ where the units of PS and surface area are μL/mL and cm^2^, respectively.

To calculate P_e_ (cm/min), the PS_e_ value was divided by the surface area (S) of the membrane:

P_e_=PS_e_/S

#### 2.8 Functional transport assay for transferrin receptors

A competitive study using transferrin and fluorescent transferrin was performed to analyze the function of transferrin receptors [21]. HBMEC/ci18 cocultured cells were incubated with human transferrin conjugated with Alexa Fluor 488 for 60 min at 37 °C. Quantification of Alexa 488-transferrin uptake and transport in an in vitro BBB model showed that the slope of the curves slightly decreased beyond 2400 pmol, suggesting that binding/uptake was saturable. To confirm ligand–receptor interaction in the in vitro human BBB model, Alexa 488-transferrin was incubated at 200 pmol with or without unlabeled transferrin at 200 pmol. Unlabeled transferrin was preincubated for 15 min.

### 2.9 Statistics

All values are presented as the means ± standard errors. Differences between two groups were tested for significance by Student's *t*-test; significant results are marked as ∗*p* < 0.05 or ∗∗*p* < 0.01.

### 2.10 Organization of SOPs

SOPs were organized with the table of contents and flow chart for their application, and provided as the supplemental information.

## 3 Results

### 3.1 Selection of benchmark items for the BBB-likeness of the BBB MPS

To characterize candidate BBB MPSs, it is necessary to have benchmark items showing BBB-likeness in order. As shown in Table 1, we selected minimal essential benchmark items based on a discussion among the pharmaceutical industry, academia, and regulators. Although astrocytes (ACs) and pericytes (PCs) modulate BBB barrier function [22,23], the basic functions originate from brain microvascular endothelial cells (BMECs). If endothelial cells are healthy and functional, they should express a sufficient amount of CD31, a marker of vascular endothelial cells. As BBB MPS performance, we should consider strong tight junction, transporter functions, and receptor mediated transcytosis. The remarkable characteristic that is not observed in the other organs is minimal paracellular transport obtained by the solid tight junction between BMECs. The tight junction is composed of transmembrane proteins, including Claudin-5 and occludin, as well as cytoplasmic adaptors such as zonula occludens protein 1 (ZO-1) [[24], [25], [26]]. As representatives, we selected Caludin-5 and ZO-1 of which expression levels and membrane localizations should be checked. The representative compounds used to confirm the practical tightness of the BBB are Lucifer Yellow and caffeine. Lucifer Yellow does not permeate BBB because of its hydrophilicity, while caffeine diffuses freely across BBB because of its lipophilicity. A lot of studies used these two compounds as references reflecting BBB tightness [[27], [28], [29], [30]]. TEER is a quick marker of high junctional tightness. The TEER value correlates with permeability of small hydrophilic molecules, although further investigation is necessary to clarify the detailed breakdown. As representative BBB transporters, we selected *P*-glycoprotein (*P*-gp), breast cancer resistant protein (BCRP) from the ATP binding cassette (ABC) superfamily and glucose transporter 1 (Glut1) from the solute carrier (SLC) superfamily. *P*-gp and BCRP contribute a lot to the drug-resistance [[32], [33], [34]], while Glut1 is the essential transporter to carry l-glucose to the CNS with high energy metabolism [[35], [36], [37]]. We added to the benchmark items the protein expression and the functions of these transporters. In the quantification of substrate transport, we suggest the use of fluorescent substrates first because of the easiness of the quantification of the fluorescence strength. However, it should be noted that rhodamine 123 and Hoechst 33,324 are not highly specific to *P*-gp and BCRP [38,39], respectively. We therefore recommend the confirmation of the results obtained in fluorescence quantification using more specific substrates: digoxin for *P*-gp, dantrolene and SASP for BCRP. The amounts of these substrates should be measured by LC-MS/MS. Furthermore, we included transferrin receptor (TfR) function as a benchmark. Receptor-mediated transcytosis (RMT) has attracted attentions of pharmaceutical companies as a mechanism to transfer of CNS-targeting drugs. Ligands or antibodies against the receptor on the luminal surface of endothelial cells undergo internalization via endocytosis and are released to the abluminal side [40,41]. TfR is one of the receptors being eagerly studied for use in RMT. We therefore added to the benchmark items the expression of TfR and the functional transport of Alexa 488-labeled transferrin.

**Table 1:**
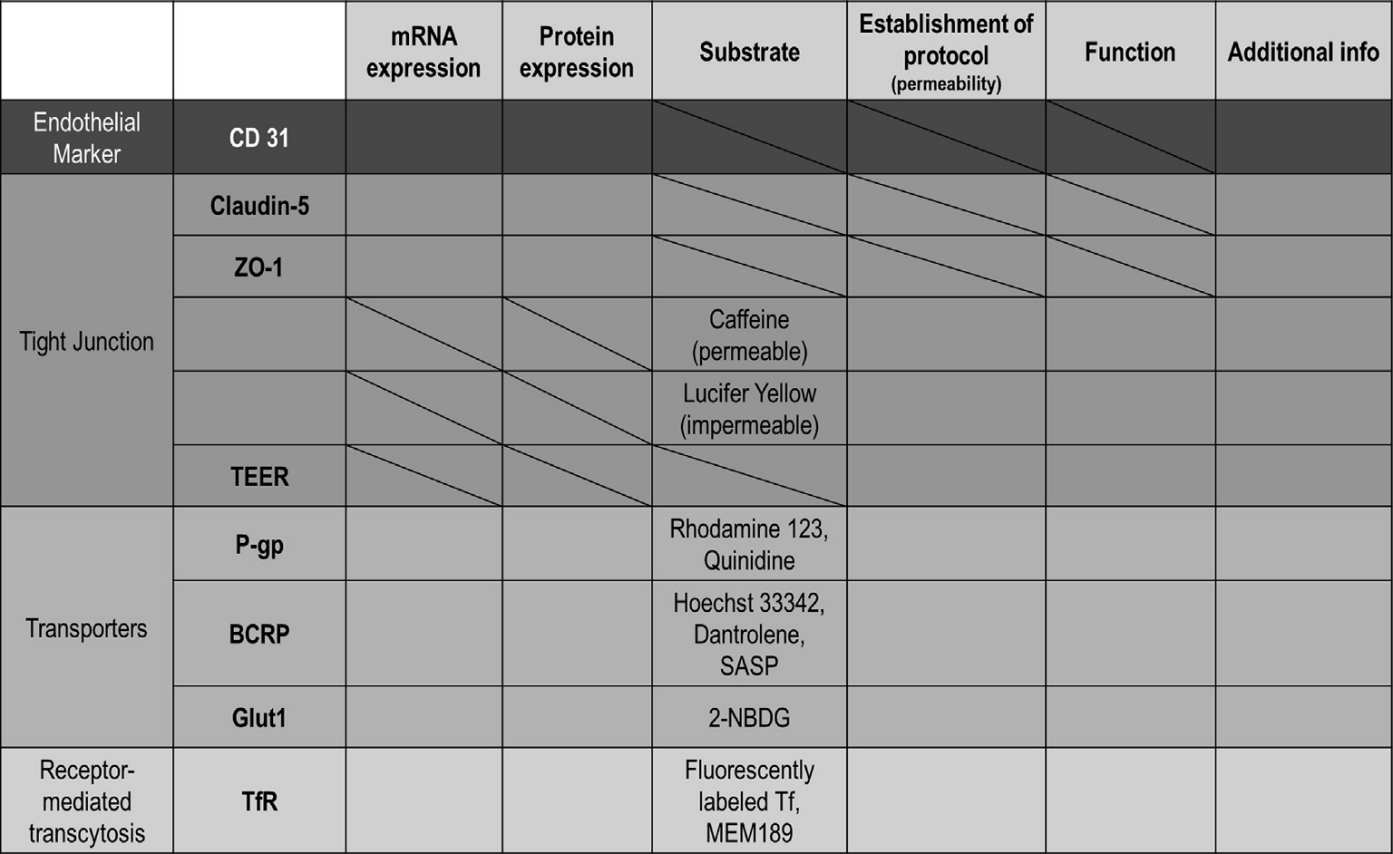
The evaluation parameters for the human in vitro BBB model.

### 3.2 Evaluation of benchmark items of the humanized tricellular static transwell BBB MPS

**Fig. 1:**
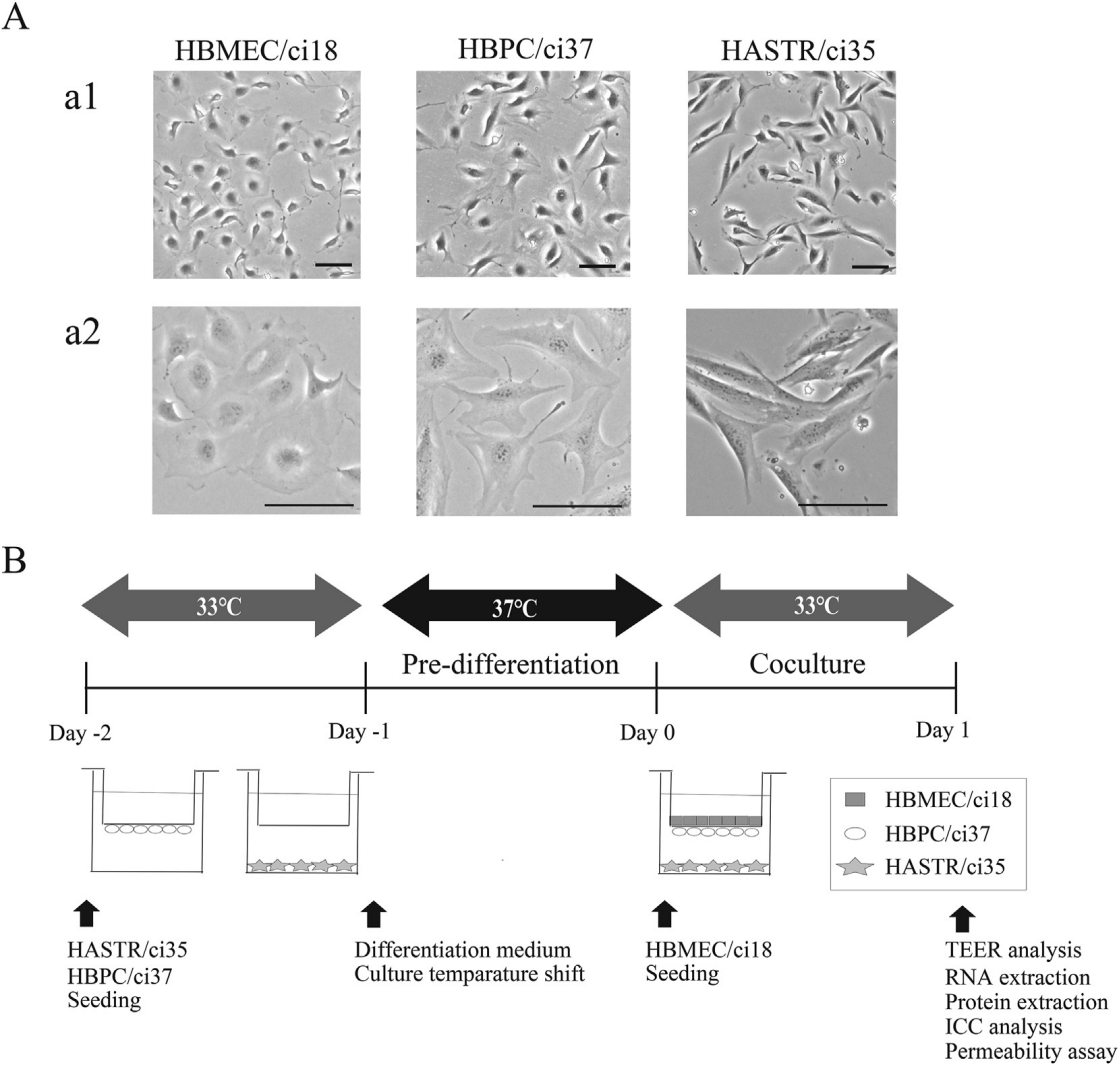
Cell morphology and schematic representation of BBB models based on immortalized human cells. A. Morphologies of HBMEC/ci18, HBPC/ci37, and HASTR/ci35 cells by phase-contrast microscopy (a1: using 4 × objective lens; a2: using 10 × objective lens; scale bar: 100 μm). B. Description of the coculture scheme.

Based on the benchmark items described above, we characterized the static transwell-type BBB MPS comprised of human immortalized BMECs (HBMEC/ci18), PCs (HBVPC/ci37), and ACs (HASTR/ci35) (Fig. 1A, a1 and 2). We preliminarily confirmed that they expressed the respective cell marker proteins (CD31 for BMECs, PDGFRβ for PCs, and aquaporin 4 for ACs) just before co-culture was initiated. The preparations of the human cell lines, the procedure for cell seeding, and the experimental schedule are shown in Fig. 1B.

**Fig. 2:**
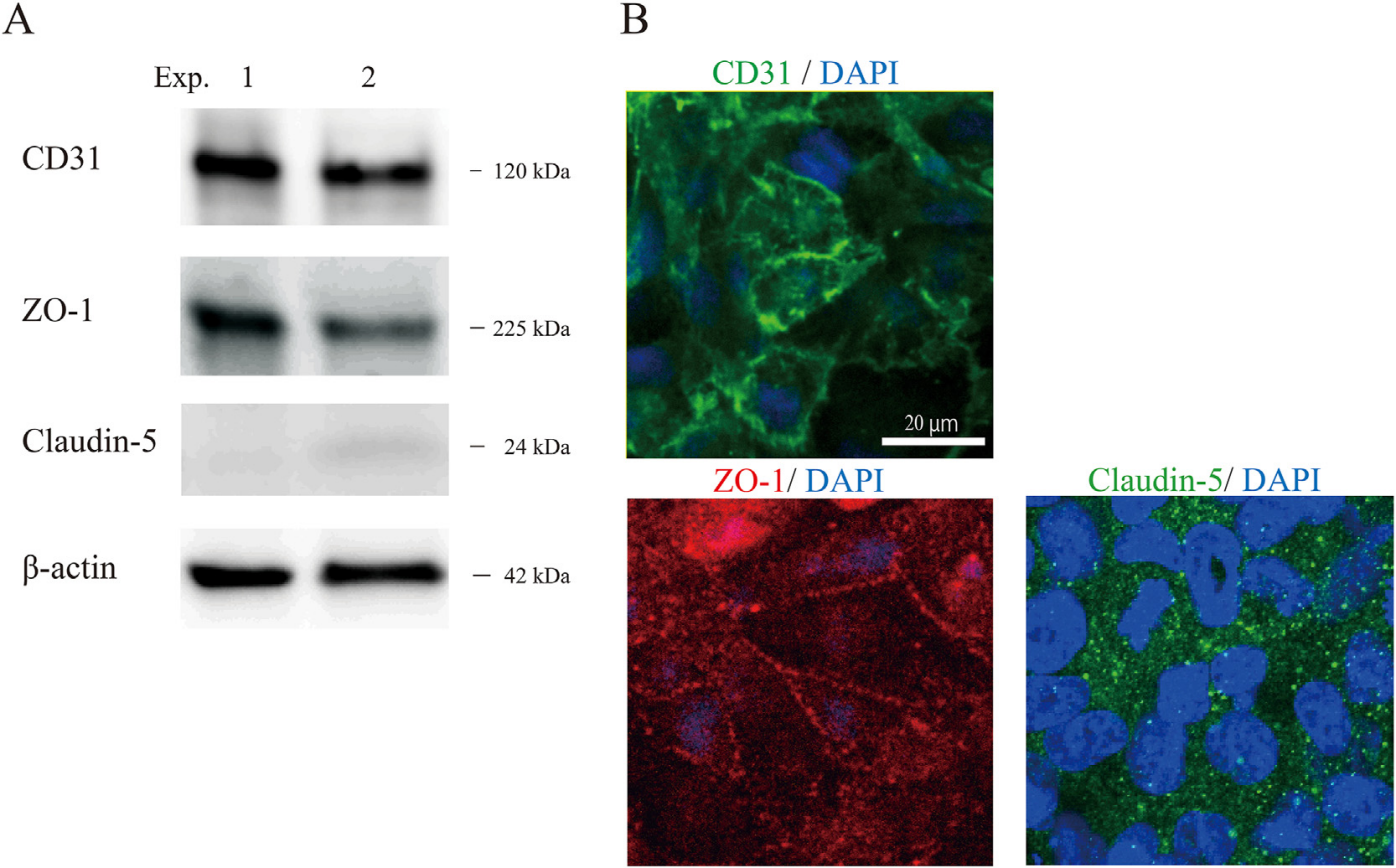
Protein expression and localization in the human in vitro BBB model. A. The expression levels of a cell marker (CD31) and two tight junction proteins (ZO-1, Claudin-5) were assessed by Western blot analysis. B. HBMEC/ci18 cells were grown on collagen IV- and fibronectin-coated insert membranes for 1 day at 37 °C and then fixed in paraformaldehyde. CD31, ZO-1, and Claudin-5 were visualized by immunocytochemistry. Scale bar: 20 μm.

**Fig. 3:**
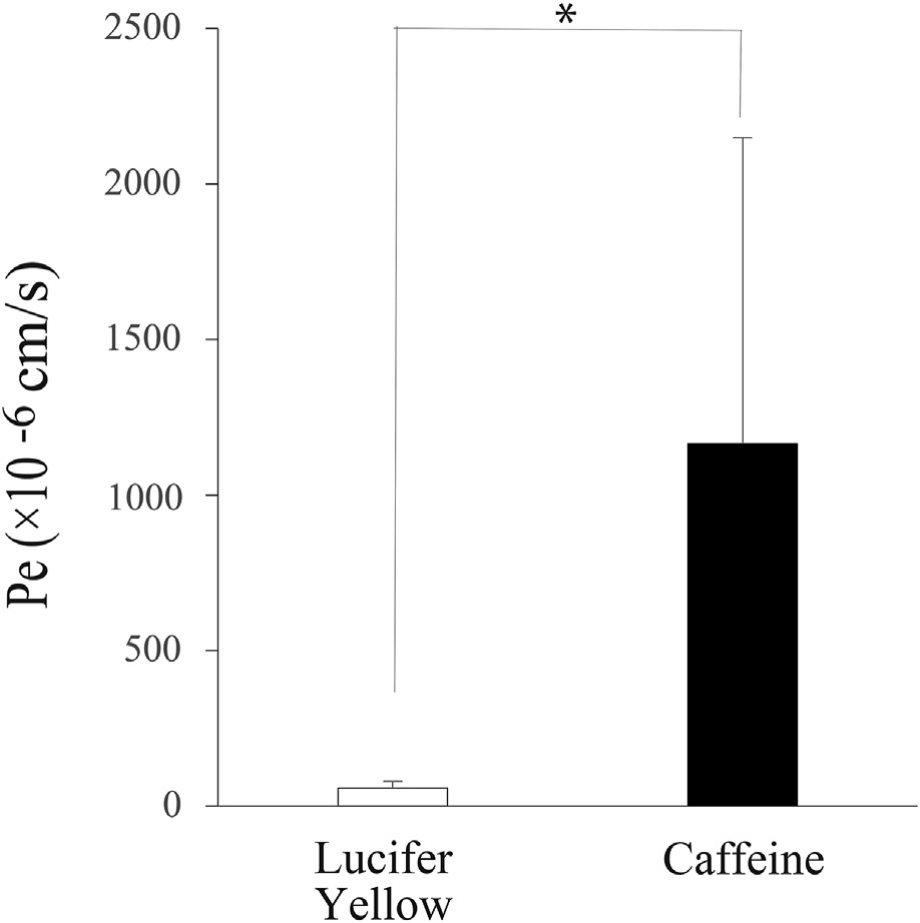
Human in vitro BBB model permeability assay using permeable and nonpermeable compounds. The permeable compound is caffeine (5 μM), and the nonpermeable compound is Lucifer Yellow (100 μM). After the addition of compounds to the apical side, the cells were incubated for 15, 30, 45, and 60 min. The medium was collected from the wells, and the concentrations of the compounds were determined. Using concentration data, the permeability coefficient (Pe) values were calculated. Each value is the mean ± S.E. of at least four independent experiments.

**Fig. 4:**
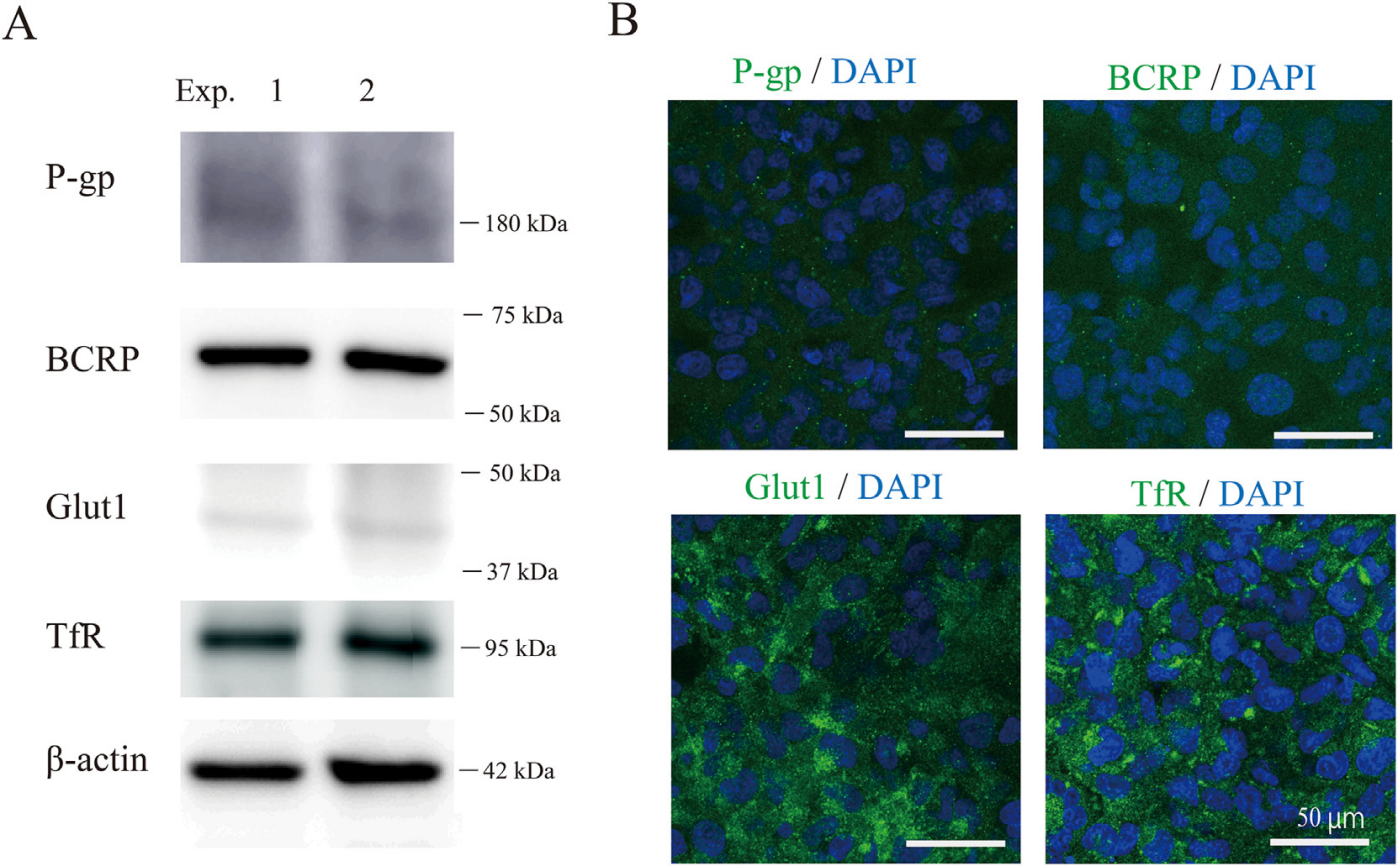
Expression and localization of transporter and receptor proteins in the human in vitro BBB model. A. *P*-gp, BCRP, Glut1, and transferrin receptor protein expression levels were assessed by Western blot analysis. B. HBMEC/ci18 cells were grown on collagen IV- and fibronectin-coated insert membranes for 1 day at 37 °C and then fixed in paraformaldehyde. *P*-gp, BCRP, Glut1, and transferrin receptor protein expression was examined by immunocytochemistry. Scale bar: 50 μm.

**Fig. 5:**
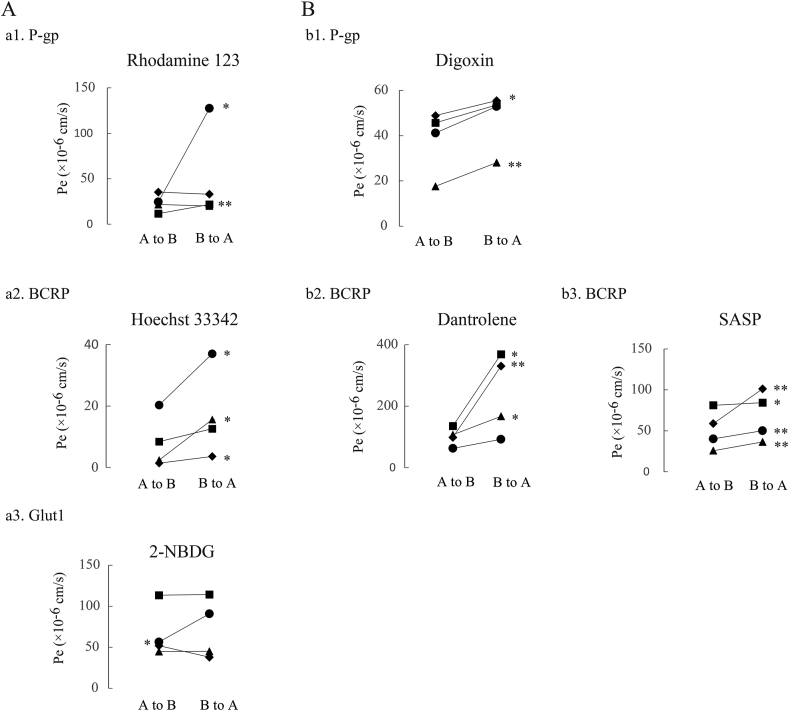
Transport function in the human in vitro BBB model. A permeability assay was performed using transporter substrates. Each plot shows the permeability coefficient (Pe) values of the transporter substrate. Each dot represents the mean value from an independent experiment. “A to B″ and “B to A″ indicate “from the apical side to the basolateral side” and “from the basolateral side to the apical side”, respectively. The concentrations were measured using a fluorescent plate reader (A) and LC‒MS/MS (B). The following concentrations were used: 10 μM for rhodamine 123, 200 μM for Hoechst 33,342, 100 μg/mg for 2-NBDG, and 5 μM for others. ∗, p < 0.05, ∗∗, p < 0.01.

**Fig. 6:**
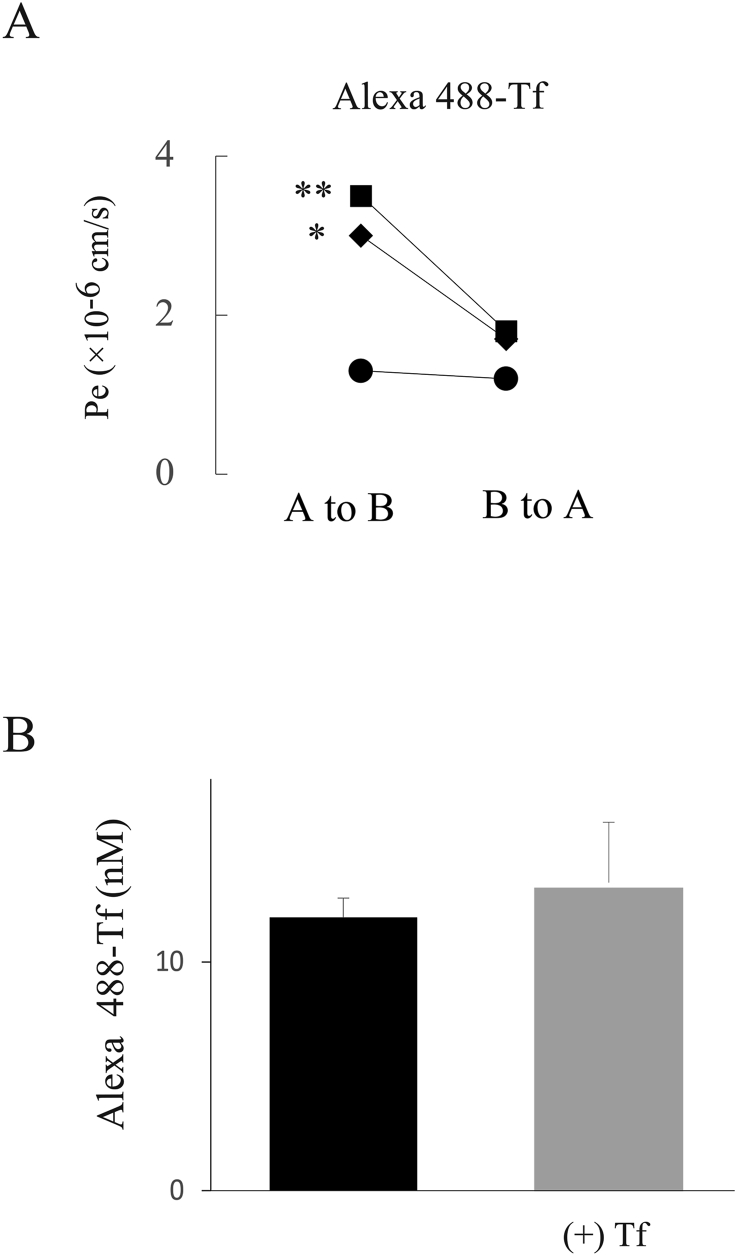
Pharmacological assay of receptor-mediated transcytosis (RMT) in the human in vitro BBB model. A. The permeability assay was performed using transporter substrates. The concentration of human transferrin (Tf) conjugated with Alexa Fluor 488 was 1 μM ∗, p < 0.05, ∗∗, p < 0.01. B. Human Alexa Fluor 488-Tf was added at 1.25 μM on the apical side, and the cells were incubated for 60 min at 37 °C with and without 15 min of preincubation with 1.25 μM nonfluorescent Tf. The transport of Alexa Fluor 488-Tf to the B side was quantified.

We first examined the expression levels of the EC marker CD31 and the TJ proteins ZO-1 and Claudin-5Claudin-5 (Fig. 2). Western blotting analysis showed a clear single band of each protein in duplicate, matching the predicted size (Fig. 2A). We also observed the cellular localization of these proteins in HBMEC/ci18 cells (Fig. 2B). CD31 was distributed in both the cell body and the cell membrane; however, stronger signals were observed on the membranes. We confirmed the membrane localization of ZO-1, while the signal of Claudin-5 was sparse and punctate. We also confirmed the consistent gene expression patterns of CD31, ZO-1, and Claudin-5 by qRT‒PCR analysis. We next investigated the basic BBB barrier integrity using BBB-permeable (caffeine) and nonpermeable (Lucifer Yellow) compounds (Fig. 3a. The P_e_ value ( × 10^−6^ cm/s) of Lucifer Yellow was 90.2 ± 23.1 (mean ± standard error), while that of caffeine was 1165.0 ± 491.7. The P_e_ values for caffeine and Lucifer Yellow were significantly different (∗: *p* < 0.05), reflecting the formation of tight junctions. We next examined the protein expression levels and cellular localizations of functional proteins, such as *P*-gp, BCRP, Glut1, and TfR (Fig. 4). Western blotting analysis detected these four proteins in duplicate, matching the predicted sizes (Fig. 4A). Immunocytochemistry showed the cellular localizations of these proteins (Fig. 4B). The signals for *P*-gp and BCRP were weak but had a discernible punctate distribution all over the cell bodies. The signals of Glut1 and TfR were stronger than those of *P*-gp and BCRP, and the expression levels of Glut1 and TfR were heterogeneous among cells. We then evaluated the functions of *P*-gp, BCRP, Glut1, and TfR. We quantified substrate transport by a bidirectional assay (Fig. 5). The apical (A)-to-basolateral (B) transfer and B-to-A transfer of the substrate in the same experiment are shown as two of the same symbol connected by a line. The raw data are shown in Table 2 (Student's *t*-test, ∗*p* < 0.05, ∗∗*P* < 0.01). To examine *P*-gp function, we checked the transport of rhodamine 123 and then digoxin. In the rhodamine 123 experiment, B-to-A transport exceeded A-to-B transport in 2 out of 4 experiments. The efflux ratio (P_e_ (B to A)/P_e_ (A to B)) of rhodamine 123 was 2.2 ± 2.0 (4 independent experiments). In the digoxin experiment, the B-to-A permeability was higher than the A-to-B permeability in 4 independent experiments. Digoxin ER was 1.3 ± 0.2 (n = 4). To examine BCRP function, we performed bidirectional transport assays with Hoechst 33,342, dantrolene, and sulfasalazine (SASP). In all experiments with these three substrates, the B-to-A permeability was higher than the A-to-B permeability (4 independent experiments). The ERs of Hoechst 33,342, dantrolene, and SASP were 2.2 ± 2.0 (n = 4), 2.2 ± 2.0 (n = 4), and 1.4 ± 0.3, respectively (n = 4). These data indicate that the efflux transporter functions of *P*-gp and BCRP were reproduced in this model. To examine Glut1 function, we used 2-NBDG as a substrate. In three out of four experiments, the A-to-B permeability and the B-to-A permeability were approximately the same. We examined TfR-mediated transcytosis using Alexa 488-labeled TF. The A-to-B permeability tended to be higher than the B-to-A permeability in three independent experiments (Fig. 6 left). To confirm whether the transport of labeled TF is mediated by TfR-mediated transcytosis, we examined the permeability of labeled transferrin in the presence and absence of unlabeled TF. Even in the presence of an equal amount of unlabeled transferrin, the amount of transported labeled TF was not changed (Fig. 6 right), suggesting that TF transport observed here was not through RMT.

**Table 2:** Summary of transport function in the human in vitro BBB model.

	Pe (10-6 cm/s)			Efflux Ratio		
	A to B	B to A			Mean	SD
Rhodamine 123	24.5	127.5	∗	5.20	2.2	2.0
	11.6	21.7	∗∗	1.87		
	35.3	33.0		0.93		
	21.6	20.1		0.93		
Hoechst 33,342	20.3	37.0	∗	1.82	3.1	2.3
	8.4	12.6		1.50		
	1.4	3.6	∗	2.57		
	2.4	15.6	∗	6.50		
2-NBDG	56.5	90.8		1.61	1.1	0.4
	113.5	114.3		1.01		
	51.9	37.9	∗	0.73		
	44.8	45.1		1.01		
Alexa 488-Transferrin	1.3	1.2		0.92	0.7	0.2
	3.5	1.8	∗∗	0.51		
	3.0	1.7	∗	0.57		
			∗P < 0.05, ∗∗P < 0.001			
	Pe (10-6 cm/s)			Efflux Ratio		
	A to B	B to A			Mean	SD
Digoxin	41.2	52.9	∗	1.28	1.3	0.2
	45.7	53.6		1.17		
	48.9	55.5		1.13		
	17.6	28.1	∗∗	1.60		
Dantrolene	62.9	92.5	∗∗	1.47	2.3	0.9
	135.5	368.9	∗	2.72		
	98.5	330.4	∗	3.35		
	107.3	166.9		1.56		
SASP	40.2	50.1	∗∗	1.25	1.4	0.3
	81.2	84.4	∗	1.04		
	58.8	101.3	∗∗	1.72		
	25.7	36.4	∗∗	1.42		
			∗P < 0.05, ∗∗P < 0.001			

A bidirectional transport assay with each substrate. Using the concentration data, the permeability coefficients Pe (A to B) and Pe (B to A), representing permeability from the apical (A) to the basolateral (B) side and vice versa, were calculated. The efflux ratio (ER) was calculated with the following equation: ER = Pe (B to A)/Pe (A to B). Each value is the mean ± S.D. from four independent experiments. More than three replicates of each experiment were performed. ∗, p < 0.05, ∗∗, p < 0.01.

### 3.3 Confirmation of the reproducibility of benchmark data in humanized tricellular static transwell BBB MPS

**Fig. 7:**
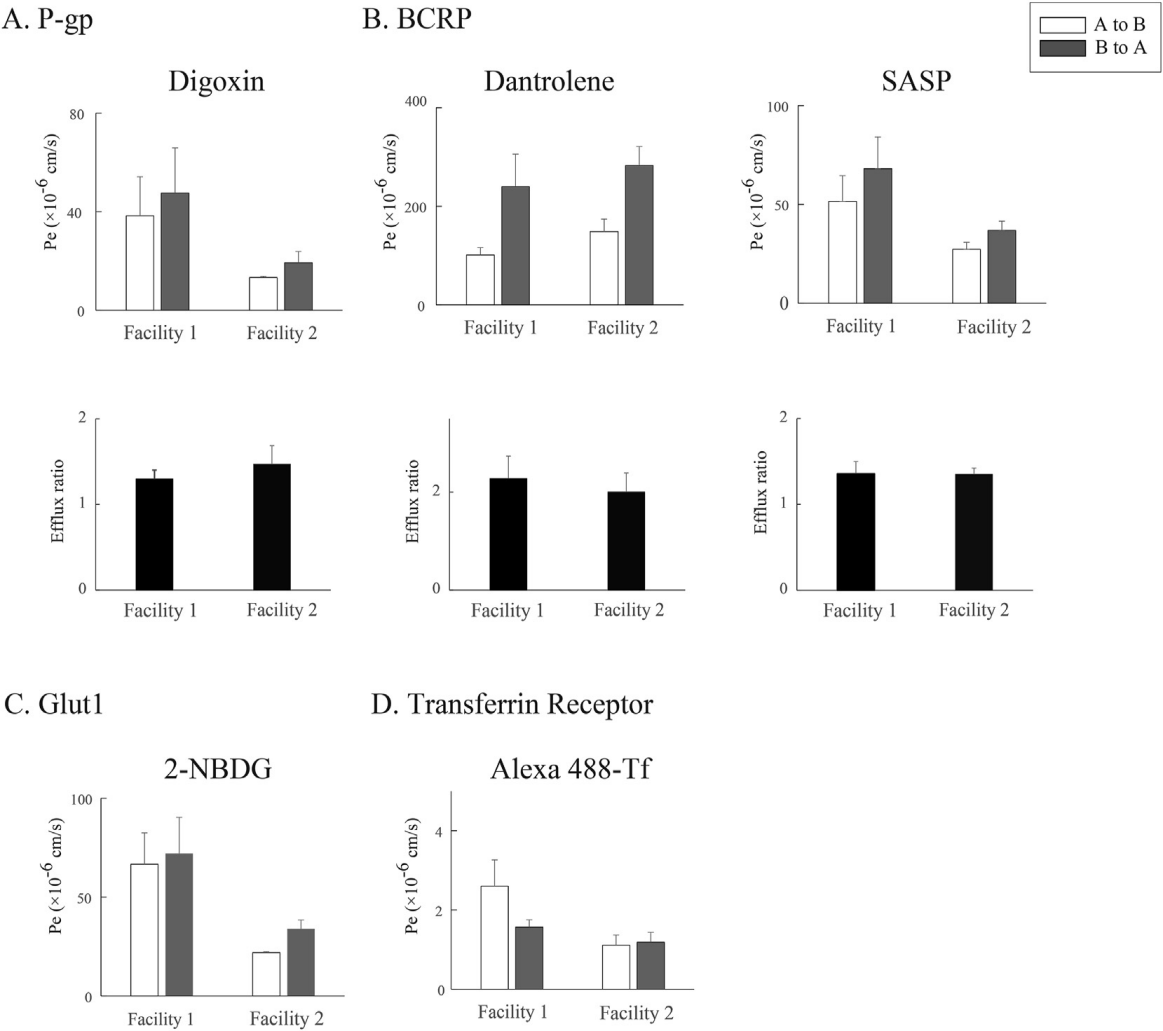
Comparison of transporter function between two public facilities. Mean A-to-B (white column) and B-to-A (gray column) Pe values. The substrates used at both facilities were digoxin (A) for *P*-gp, dantrolene (b1) and SASP (b2) for BCRP, 2-NBDG (C) for Glut1, and Alexa Fluor 488-Transferrin (D) for the transferrin receptor. (Transferrin: n = 3 at each facility; all others: n = 4 at Facility 1, n = 3 at Facility 2).

We obtained the data suggesting the humanized tricellular static transwell BBB MPS is suitable to investigate the interaction of new drugs with *P*-gp and BCRP. Therefore, another facility in our project (Facility 2 in the graphs) performed the bidirectional transport assay according to the same SOP and compared the data with ours (Facility 1in the graphs) to confirm the data reproducibility (Fig. 7). Concerning the *P*-gp function, the Efflux ratio of Digoxin was 1.3 ± 0.2 (n = 4) in Facility 1 and 1.5 ± 0.4 (n = 3) in Facility 2 (Fig. 7A). Concerning the BCRP function, the ER of Dantrolene was 2.2 ± 2.0 (n = 4) in Facility 1 and 2.0 ± 0.7 (n = 3) in Facility 2 (Fig. 7B, left). The ER of SASP was 1.4 ± 0.3 (n = 4) in Facility 1, and 1.4 ± 0.1 (n = 3) in Facility 2 (Fig. 7B, right). The ER values for *P*-gp and BCRP obtained in two facilities were almost same, indicating reproducibility of the data and robustness of the SOP. On the other hand, directional transports mediated through Glut1 or TfR were not confirmed in two-facility experiments. The comparison data between two facilities indicate that a human immortalized cell-based tricellular static BBB MPS is suitable to evaluate the *P*-gp and BCRP functions, but not so suitable to evaluate Glut1 and TfR.

## 4 Discussion

In this study, we organized the minimal essential benchmark items reflecting ‘BBB-likeness’ of BBB MPS and SOPs of these evaluation. This is the important developmental step for the social acceptance of BBB MPS, because these benchmarks support end users in checking the performance and the application range of the BBB MPS. We performed trial run using a 2D humanized tricellular static transwell BBB MPS and concluded that this BBB MPS are appropriate for functional assays of *P*-gp or BCRP, not for those of Glut1 or TfR.

Previous reports have shown that this type of tricellular static transwell BBB MPS resulted in tight junctions and efflux transporter function [11]. We re-characterized this model based on the BBB MPS benchmark items that had been determined in this study. We obtained data consistent with a previous report [11]. In this study, we also examined the expression level and cellular localization of Claudin-5. Even if single bands were detected by Western blotting, the expression level was low, and clear membrane localization was not observed by immunocytochemistry. In a past comparative study, the expression level of Claudin-5 was found to be lower in HBMEC/ci18 than in primary cultured HBMECs [42]. Claudin-5 is the dominant tight junction protein in BMECs [43,44]; however, recent studies indicated an inverse correlation between Claudin-5 and TJ functionality [45,46]. Therefore, we did not use the expression level of Claudin-5 as a proxy for TJ functionality but only as a marker for TJs.

In this study, we (facility 1) and the Stem Cell Evaluation Technology Research Association (facility 2) confirmed the reproducibility of the SOPs and data of the functional assays for *P*-gp, BCRP, Glut1, and TfR, which had been selected as benchmark items to confirm that a system has the degree of BBB-likeness necessary for drug development. In bidirectional transport assay, directional Glut1 transport and TfR-mediated transcytosis were not confirmed in the two-facility experiments. It might be difficult to reproduce the cell polarity-related functions in two dimensional model like this. In support of this, Kitamura et al. showed remarkable 2-NBDG uptake in the multicellular spheroidal BBB model comprised of the immortalized human BBB cell lines [47]. In addition, the expression levels of Glut1 and TfR were increased [48] and TfR-mediated RMT was detected in three dimensional model in which brain microvasculature network was reproduced in the fibrin gel [49]. On the contrary, the function data of *P*-gp and BCRP showed high reproducibility. These data are of use for various stages of drug development. For example, in case of compounds targeting intracranial neoplasms, penetration is limited by *P*-gp and BCRP [50,51] expressed in brain tumors [52,53]. The transporter assay using this BBB MPS can be applied to determine the concentrations of antitumor drugs and co-applied efflux transporter inhibitors.

In summary, we have identified a set of benchmark items to assess the BBB-likeness of BBB MPS. As a result of the benchmark assay of the 2D humanized tricellular static transwell BBB MPS, we showed that this BBB MPS are appropriate for functional assays of *P*-gp or BCRP, suggesting the SOPs organized in this study are robust enough to check the performance and appreciation range of the candidate BBB MPS.

## Declaration of competing interest

All authors declare no conflicts of interest.
