# Supplementary material for: Usefulness of a humanized tricellular static transwell blood–brain barrier model as a microphysiological system for drug development applications. - A case study based on the benchmark evaluations of blood-brain barrier microphysiological system
Source: Regen Ther. 2023 Feb 24;22:192–202. doi: 10.1016/j.reth.2023.02.001 (PMC9988422; doi:10.1016/j.reth.2023.02.001)
Supplement: Multimedia component 1 [file mmc1.pptx]

## Slide 1
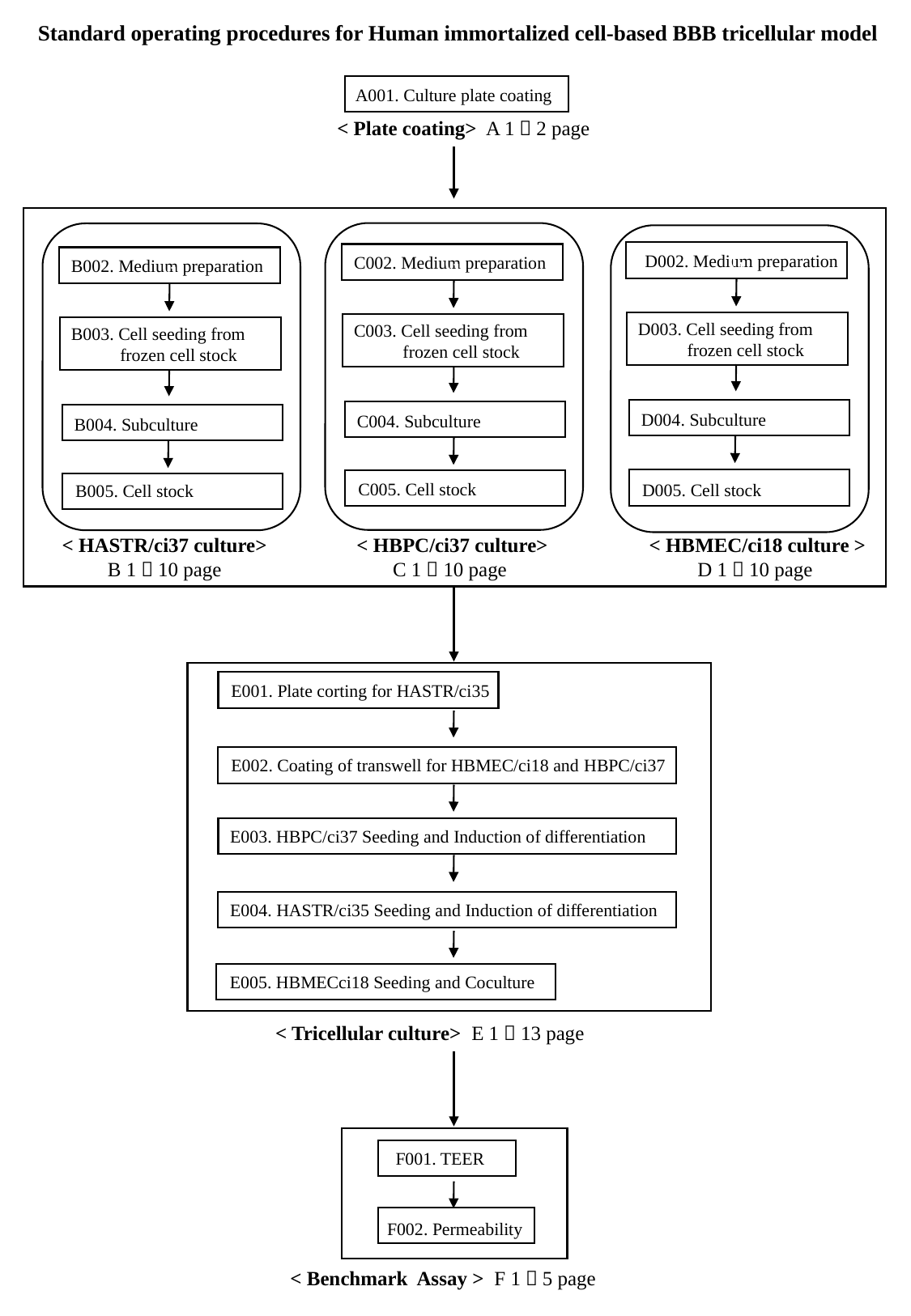

Standard operating procedures for Human immortalized cell-based BBB tricellular model
A001. Culture plate coating
G
D002. Medium preparation
G
C002. Medium preparation
G
B002. Medium preparation
D003. Cell seeding from
 frozen cell stock
C003. Cell seeding from
 frozen cell stock
B003. Cell seeding from
 frozen cell stock
D004. Subculture
C004. Subculture
B004. Subculture
D005. Cell stock
C005. Cell stock
B005. Cell stock
< HASTR/ci37 culture> B 1～10 page
< HBPC/ci37 culture>
C 1～10 page
< HBMEC/ci18 culture >
D 1～10 page
E001. Plate corting for HASTR/ci35
E002. Coating of transwell for HBMEC/ci18 and HBPC/ci37
E003. HBPC/ci37 Seeding and Induction of differentiation
E004. HASTR/ci35 Seeding and Induction of differentiation
E005. HBMECci18 Seeding and Coculture
< Tricellular culture> E 1～13 page
F001. TEER
F002. Permeability
< Plate coating> A 1～2 page
< Benchmark Assay > F 1～5 page
