# Supplementary material for: Usefulness of a humanized tricellular static transwell blood–brain barrier model as a microphysiological system for drug development applications. - A case study based on the benchmark evaluations of blood-brain barrier microphysiological system
Source: Regen Ther. 2023 Feb 24;22:192–202. doi: 10.1016/j.reth.2023.02.001 (PMC9988422; doi:10.1016/j.reth.2023.02.001)

**Contents of standard operating procedures**

**A. Plate coating**

A001. Culture plate coating for three types of Human Immortalized cells1

**B. HASTR/ci37 culture**

B001．Medium preparation 1

B002．Cell seeding from frozen cell stock 3

B003．Subculture 5

B004．Cell stock 8

**C. HBPC/ci37 culture**

C001．Medium preparation 1

C002．Cell seeding from frozen cell stock 3

C003．Subculture 5

C004．Cell stock 8

**D. HASTR/ci37 culture**

D001．Medium preparation 1

D002．Cell seeding from frozen cell stock 3

D003．Subculture 5

D004．Cell stock 8

**E. Tricelluar Culture**

E001．Plate coating for HASTR/ci35 1

E002．Coating of transwell for HBMEC/ci18 and HBPC/ci37 3

E003．HBPC/ci37 Seeding and Induction of differentiation 5

E004．HASTR/ci35 Seeding and Induction of differentiation 8

E005．HBMEC/ci18 Seeding and Coculture 11

**F. Benchmark Assay**

F001．TEER Measurement 1

F002．Permeability and bidirectional Transport assay 3

**A001．Culture plate coating for three types of Human Immortalized cells**

- Date & Time： Y M D（ ） ： ～ ：
- Operator：（ ）
- Room Temperature： ℃

＊How to coat dishes with collagen type I（Common to three type of cells）

＊Commercial pre-coated plates（IWAKI, #4020010）

| No | Process | Note |
| --- | --- | --- |
| A001.1 | Preparation and confirmation of necessary items  〇Equipment：  Clean bench, Electric pipettor, Pipette  〇Supplies：  Pipettes、Pipette Tips, 50mL tube  〇Reagents：  □Cellmatrix Type I-C 3㎎/mL 100mL (Nitta gelatin)  □Media for each cell  □Dilute hydrochloric acid（1mM, 0.001N）  5N（5mol/L） HCL（Wako, 081-05435）After measuring 100 µL with ultrapure water to make 500 mL, autoclave sterilization.  〇Culture dishes：  □φ100mm dish ( ）  □φ60mm dish ( ） | All equipment should be sterilized with 70% ethanol and placed in a safety cabinet.  Dilute hydrochloric acid: Filter sterilization of φ0.2µm pore is also possible. |
| A001.2 | Prepare the required amount of collagen solution. (Cellmatrix Type I-C is diluted 30-fold with dilute hydrochloric acid.)  ・φ100mm dish  5mL × dish ＝ A mL  ・φ60mm dish  3mL × dish ＝ B mL  Required amount of Cellmatrix Type I-C solution    A＋B mL /30 ＝ C mL  Required amount of hydrochloric acid  A＋B mL - C mL ＝D mL | Collagen solution: 0.1mg/mL |
| A001.3 | Add collagen solution to culture dish  ・φ100mm dish 5mL/dish  ・φ60mm dish 3mL/dish |  |
| A001.4 | Let the dishes stand in the clean bench for 15minutes. |  |
| A001.5 | Aspirate and remove the collagen solution. |  |
| A001.6 | Open the lid and let it dry. (1 to 2 hours)  ： ～ ：  UV irradiation for 15 minutes  ： ～ ： |  |
| A001.7 | After drying, fill in the "date" and "worker name", wrap it, and store it in the clean bench. | Can be stored for 1 to 2 months.  On the day of cell seeding, wash twice with culture medium before use, and then add the cell suspension. |
| - MEMO | | |

※No: Inside the Clean bench．

**B001．HASTR/ci37 Culture Medium preparation**

- Date & Time： Y M D（ ） ： ～ ：
- Operator：（ ）
- Room Temperature： ℃

| No | Process | Note |
| --- | --- | --- |
| B001.1 | Preparation and confirmation of necessary items  〇Equipment：  Clean bench, Electric pipettor, Pipette  〇Supplies：  Pipettes, Pipette Tips, 50mL tube, Sterilized storage bottle  〇Reagents：   - Astrocyte basal Medium (Gibco, A1261301)   ・DMEM＋Glutamax 500mL (4℃） (Gibco, 10569-010）  ・One shot FBS 50mL (-20℃） (Gibco, A31604-01）  ・N2 supplement 5mL (-20℃） (Gibco, 17502-048）   - Penicillin/streptomycin（Gibco, 15140-122） - cAMP (Sigma, A6885)   Final concentration: 1mM  Stock solution: Prepared with dH_2_O to 100mM. 0.22µm filter sterilization. Stored at -20°C.  Date：   - Blasticidin S Hydrochloride（Wako, 029-18701）   Stock solution: Prepared at 4mg/mL (1000X) with dH_2_O. 0.22µm filter sterilization. Storage at 4°C. Use within 1month.  Date： | All equipment should be sterilized with 70% ethanol and placed in the clean bench.  Use up the medium supplement by the expiration date. 　Supplement can be re-frozen up to once.  cAMP, Blasticidin S: Avoid freeze/thaw cycles. |
| B001.2 | The following reagents are mixed to prepare the astrocyte basal medium.  **DMEM+Glutamax**  176mL  FBS 20mL  N2 supplement 2mL  Penicillin/streptomycin 2mL  200mL  Date： | Medium cannot be frozen. |
| B001.3 | Move the bottle horizontally in a circular motion to stir. Fill in the bottle with "Astrocyte basal medium", "Date" and "Worker name". Store at 4°C. | Avoid foaming when stirring.  Store at 4°C and use within 2 weeks. |
| B001.4 | Preparation of astrocyte passage medium  Astrocyte basal medium 50mL  Blasticidin S 4 mg/mL stock (1000X) 50µL  Date： | Use up the medium supplement by the expiration date. 　Supplement can be re-frozen up to once.  To prevent Blasticidin S from deactivating, small volume preparation at once is recommended. |
| B001.5 | Mix by inverting. Fill in "Astrocyte passage medium", "Date" and "Worker name". Store at 4°C. |  |
| B001.6 | Preparation of Astrocyte differentiation medium  **DMEM＋Glutamax** 48.95mL  　N2 supplement 0.5mL  　Penicillin/streptomycin 0.5mL  cAMP solution (100mM) 0.05mL  50mL    Date： | Make the required amount.  Prepare at the time of use.  A medium in which serum is removed from the astrocyte passage medium and cAMP is added. |
| B001.7 | Mix by inverting. Fill in the bottle with "Astrocyte basal medium", "Date" and "Worker name". Store at 4°C. |  |
| - MEMO | | |

※No: Inside the clean bench

**B002．****Culture procedure for HASTR/ci35 Cell seeding from frozen cell stock**

- Date & Time： Y M D（ ） ： ～ ：
- Operator：（ ）
- Room Temperature： ℃

| No | Process | Note |
| --- | --- | --- |
| B002.1 | Preparation and confirmation of necessary items  〇Equipment：  Water bath, Clean bench, Centrifuge, Electric pipettor, Timer Clock, Dewar bottle for Liquid Nitrogen, Tweezers, Cell counter, Liquid nitrogen storage tank, Pipette, CO_2_ incubator（33℃, 5% CO_2_）  *Warm water bath at 37℃  〇Supplies：  Pipettes, Pipette Tips, 50mL tube, 15mL tube, Sterilized storage bottle, φ100mm dish (Collagen type I-coated), Hemocytometer  〇Reagents：  □Astrocyte passage medium(Store at 4℃)  DMEM＋Glutamax, 1% N2 supplement, 10% FBS,  1% Penicillin/streptomycin, 4µg/mL Blasticidin S  〇Cell： HASTR/ci35  Passage # , (1.0x10^6^ cells/vial)  Date , Lot | All equipment should be sterilized with 70% ethanol and placed in the clean bench.  Collagen type I coating（Refer to 001）  Use within 2weeks. (Refer to A002） |
| B002.2 | Astrocyte passage medium to warm to the room temperature.  ： ～ ： |  |
| B002.3 | Dispense the required amount (about 10 mL/tube) of astrocyte passage medium into a conical tube. |  |
| B002.4 | Write the "cell name", "passage number", "worker name", and "date" on the lid of the Collagen type I-coated φ100mm dish |  |
| B002.5 | Remove the frozen cell stock from the liquid nitrogen cell storage tank and move it in a Dewar bottle. |  |
| B002.6 | Take out the cryotube with tweezers, and warm it in a 37°C water bath while shaking. | Avoid getting water on the lid to prevent contamination.  Thaw frozen fluid to the extent that it moves away from the wall. |
| B002.7 | The cryotube should be sterilized with 70% ethanol and placed in the clean bench. |  |
| B002.8 | Transfer cells from cryotube to conical tube pre-dispensed with medium |  |
| B002.9 | Rinse the cryotube with cell suspension and return to the conical tube. |  |
| B002.10 | Centrifuge the conical tube at 120xg for 3min. |  |
| B002.11 | Remove the supernatant with an ejector to the extent that the cells are not exposed, and loosen the cell pellet by tapping. |  |
| B002.12 | Add 10 mL of Astrocyte passage medium along tube wall |  |
| B002.13 | Slowly suspend by pipetting with a 10mL pipette. | Vortex strictly prohibited. |
| B002.14 | Wash φ100mm dish (Collagen type I coated)（B002.4）with Astrocyte passage medium twice and seed cells. | No need washing in the case of pre-cort products. |
| B002.15 | Incubate cells at 33℃ with 5% CO_2._  ： ～ ： | 70-80% confluent for 2-3 days |
| ●MEMO | | |

※No: Inside the safety cabinet

**B003．Culture procedure for HASTR/ci35 Subculture**

- Date & Time： Y M D（ ） ： ～ ：
- Operator：（ ）
- Room Temperature： ℃

| No | Process | Note |  |
| --- | --- | --- | --- |
| B003.1 | Preparation and confirmation of necessary items  〇Equipment：  Water bath, Clean bench, Centrifuge, Electric pipettor, Timer Clock, Pipette, CO_2_ incubator（33℃, 5% CO_2_）  〇Supplies：  Pipettes, Pipette Tips, 50mL tube, 15mL tube, Sterilized storage bottle, φ100mm dish (Collagen type I-coated), Hemocytometer  φ100mm/φ150mm dish (Collagen type I-coated)  〇Reagents：  □Astrocyte passage medium (Store at 4°C)  DMEM＋Glutamax, 1% N2 supplement, 10% FBS,  1% Penicillin/streptomycin, 4µg/mL Blasticidin S  □PBS(-) (Gibco, 14190-144)  □0.25% Trypsin-EDTA (1×) (Gibco, 25200-056)  Dilute to 0.1% Trypsin-EDTA with PBS (-).  □0.4% Trypan blue solution (SIGMA, T8154)  〇Cell： HASTR/ci35  Passage # , (1.0x10^6^ cells/vial)  Date , Lot | All equipment should be sterilized with 70% ethanol and placed in the clean bench.  Collagen type I coating（Refer to 001）  Or use the pre-coating products (IWAKI, #4020-010）  Use within 2 weeks（Refer to A002）  Observe the expiration date of the medium. |  |
| B003.2 | Astrocyte passage medium to warm to the room temperature.  ： ～ ： |  |  |
| B003.3 | Dilute 0.25% Trypsin-EDTA mL with mL PBS (-) to make 0.1% Trypsin-EDTA. |  |  |
| B003.4 | Remove HASTR / ci35 cells from the 33°C 5% CO2 incubator and observe them. | Make sure that the cell confluency is 80% or more. |  |
| B003.5 | Remove the culture medium of HASTR / ci35 cells by suction. |  |  |
| B003.6 | Wash HASTR/ci35 cells with PBS (-).  PBS volume: φ100mm dish (5-10mL), φ150mm dish (20-30mL) |  |  |
| B003.7 | Remove PBS (-) by suction and add 0.1% Trypsin-EDTA (B003.3) to spread throughout the cells.  Trypsin volume: φ100mm dish (2mL), φ150mm dish (5mL) |  |  |
| B003.8 | Transfer the dish to a 33°C 5% CO_2_ incubator and warm for 3-4 minutes.  ： ～ ： |  |  |
| B003.9 | Tap the side of dish with the lid on, and confirm visually and microscopically that the cells have peeled off. |  |  |
| B003.10 | Add astrocyte passage medium. Adjust the amount of medium according to the dilution rate (see below). Volume: φ100mm dish (7-8mL), φ150mm dish (15-25mL) | Inactivate trypsin by FBS in medium |  |
| B003.11 | Slowly resuspend with an electric pipettor. | Vortex and intense suspension are strictly prohibited. |  |
| B003.12 | Collect the cell suspension in a conical tube.  Cell suspension（A ）mL |  |  |
| B003.13 | Take a potion of the suspension of B003.12 and mix with the trypan blue solution by pipetting.  Cell suspension: Trypan blue solution = 1:1  … 2-fold dilution (*) |  |  |
| B003.14 | Add the suspension to the hemocytometer and count the cell number of four corners on the hemocytometer under a microscope.  B＝（ ＋ ＋ ＋ ）÷4×2（*）  Cell density  ＝[B ]×10^4^ cells/mL  ＝[C ]×10^6^ cells/mL  total cell number  [C ]×10^6^ cells/mL× [A ] mL  ＝[D ]×10^6^ cells | Count cells without dyed with trypan blue as living cells. |  |
| B003.15 | Write the "cell name", "passage number", "worker name", and "date" on the lid of the Collagen type I-coated φ100mm dish  Medium Volume: φ100mm dish (5-10mL), φ150mm dish (20-30mL) | No need washing in the case of pre-coating products. |  |
| B003.16 | After removing the wash medium from the Dish, add an amount of astrocyte passage medium according to the dilution rate and re-seed the HASTR/ci35 cell suspension of B003.12. (Dilution rate: 1:2 to 1:6).  Total medium volume per dish: φ100mm (10mL), φ150mm (30mL)  Record the dilution rate and the number of dishes. | Cell density at 1×10^6^ cells/dish (100mm) or more.  Doubling time: 48-72hr |  |
| B003.17 | Write the "cell name", "Astrocyte passage number", "worker name", and "date" on the lid of the Collagen type I-coated φ100mm dish |  |  |
| B003.18 | Incubate cells at 33°C with 5% CO_2._  ： ～ ： | 70-80% confluent for 3-4 days. |  |
| - MEMO | | | |

※No: Inside the safety cabinet

**B004. Culture procedure for HASTR/ci35 Cell stock**

- Date & Time： Y M D（ ） ： ～ ：
- Operator：（ ）
- Room Temperature： ℃

| No | Process | Note |
| --- | --- | --- |
| B004.1 | Preparation and confirmation of necessary items  〇Equipment：  Water bath, Clean bench, Centrifuge, Electric pipettor, Timer Clock, Pipette, CO_2_ incubator（33℃, 5% CO_2_）, Dewar bottle for Liquid Nitrogen, Tweezers, Cell counter, Liquid nitrogen storage tank  〇Supplies：  Pipettes, Pipette Tips, 50mL tube, 15mL tube, Sterilized storage bottle, φ100mm dish (Collagen type I-coated), Hemocytometer,  Cryotube:  〇Reagents：  □Astrocyte passage medium (Store at 4°C)  DMEM＋Glutamax, 1% N2 supplement, 10% FBS,  1% Penicillin/streptomycin, 4µg/mL Blasticidin S  □PBS(-)(Gibco, 14190-144)  □0.25% Trypsin-EDTA (1×) (Gibco, 25200-056)  Dilute to 0.1% Trypsin-EDTA with PBS (-).  □0.4% Trypan blue solution (SIGMA, T8154)  □Banbanker (Nippon genetics, CS-04-001)  〇Cell： HASTR/ci35  Passage # , (1.0x10^6^ cells/vial)  Date , Lot | All equipment should be sterilized with 70% ethanol and placed in a clean bench  Cryotube:  Any tube that can be used for storage in liquid nitrogen  Use within 2weeks. （Refer to A002）. |
| B004.2 | Astrocyte passage medium to warm to the room temperature.  ： ～ ： |  |
| B004.3 | Dilute 0.25% Trypsin-EDTA mL with mL PBS (-) to make 0.1% Trypsin-EDTA. |  |
| B004.4 | Remove HASTR/ci35 cells from the 33°C 5% CO2 incubator and observe them. | Make sure that the cell confluency is 80% or more. |
| B004.5 | Remove the culture medium of HASTR/ci35 cells by suction. |  |
| B004.6 | Wash HASTR/ci35 cells with PBS (-).  PBS volume: φ100mm dish (5-10mL), φ150mm dish (20-30mL) |  |
| B004.7 | Remove PBS (-) by suction and add 0.1% Trypsin-EDTA (B003.3) to spread throughout the cells.  Trypsin volume: φ100mm dish (2mL), φ150mm dish (5mL) |  |
| B004.8 | Transfer the dish to a 33°C 5% CO2 incubator and warm for 3-4 minutes.  ： ～ ： |  |
| B004.9 | Tap the side of dish with the lid on, and confirm visually and microscopically that the cells have peeled off. |  |
| B004.10 | Add astrocyte passage medium.  Volume: φ100mm dish (8ml), 150mm dish (20ml) | Inactivate trypsin by FBS in medium |
| B004.11 | Slowly resuspend with a pipettor. | Vortex and intense suspension are strictly prohibited. |
| B004.12 | Collect the cell suspension in a conical tube.  Cell suspension（A ）mL |  |
| B004.13 | Take a portion of the suspension of B003.12 and mix with the trypan blue solution by pipetting.  Cell suspension: Trypan blue solution = 1:1  … 2-fold dilution (*) |  |
| B004.14 | Add the suspension to the hemocytometer and count the cell number of four corners on the hemocytometer under a microscope.  B＝（ ＋ ＋ ＋ ）÷4×2（＊）  Cell density  ＝[B ]×10^4^ cells/mL  ＝[C ]×10^6^ cells/mL  total cell number  [C ]×10^6^ cells/mL× [A ] mL  ＝[D ]×10^6^ cells  Resuspend in a [D ] mL Banbanker. | Count cells without dyed with trypan blue as living cells.  Make the stock 1×10^6^cells/tube/mL |
| B004.15 | Centrifuge the conical tube at 120xg for 3min. |  |
| B004.16 | Remove the supernatant by suction, add a [D ] mL Banbanker, and slowly suspend with an electric pipettor. |  |
| B004.17 | Place 1 mL/tube of Banbanker cell suspension (B004.16) in a cryotube and lid tightly. | The lid is sterilized by heating |
| B004.18 | Write the "cell name", "passage number", "Cell density” "worker name", and "date" on the tube.  Place in a cell freezing container and freeze at -80°C for at least half a day.  ： ～ ： |  |
| B004.19 | Transfer the frozen cell stock to a liquid nitrogen cell storage tank.  Date： / | After freezing at -80°C transfer as soon as possible. |
| - MEMO | | |

※No: Inside the clean bench

**C001．Culture procedure for HBPC/ci37 Preparation for medium**

- Date & Time： Y M D（ ） ： ～ ：
- Operator：（ ）
- Room Temperature： ℃

| No | Process | Note |
| --- | --- | --- |
| C001.1 | Preparation and confirmation of necessary items  〇Equipment：  Clean bench, Electric pipettor, Pipette  〇Supplies：  Pipettes, Pipette Tips, 50mL tube, Sterilized storage bottle  〇Reagents：  □Pericyte Medium (ScienCell, 1201)  ・Basal Medium 500mL （4℃）  ・FBS 10mL（-20℃）  ・Pericyte Growth Supplement 5mL（-20℃）  ・Penicillin/streptomycin 5mL（-20℃）  □FBS, Pericyte Growth Supplement, Penicillin/streptomycin  Dispense 1mL each at Store at -20°C.  Date：  □Blasticidin S Hydrochloride（Wako, 029-18701）  Stock solution: Prepared at 4mg/mL (1000X) with dH_2_O. 0.22µm filter sterilization. Storage at 4°C. Use within 1month.  Date： | All equipment should be sterilized with 70% ethanol and placed in the clean bench  Use up the medium supplement by the expiration date. 　Supplement can be re-frozen up to once.  Medium cannot be frozen.  Use within 1 month.  Blasticidin S: Avoid freeze/thaw cycles. |
| C001.2 | The following reagents are mixed to prepare the Pericyte basal medium.  Basal Medium 192mL  FBS 4mL  Pericyte Growth Supplement 2mL  Penicillin/streptomycin 2mL  200mL  Date： |  |
| C001.3 | Move the bottle horizontally in a circular motion to stir. Fill in the bottle with "Pericyte basal medium", "Date" and "Worker name". | Store at 4°C and use within 1month. |
| C001.4 | Preparation of Pericyte passage medium  Pericyte basal medium 50mL  Blasticidin S 4mg/mL stock (1000X) 50µL  Date： | Use up the medium supplement by the expiration date. 　Supplement can be re-frozen up to once.  To prevent Blasticidin from deactivating, small volume preparation at once are recommended. |
| C001.5 | Mix by inverting. Fill in "Pericyte passage medium", "Date" and "Worker name". | Store at 4°C and use within 1month. |
| C001.6 | Preparation of Pericyte differentiation medium  Basal Medium 49mL  Pericyte Growth Supplement 0.5mL  Penicillin/streptomycin 0.5mL  Date： | Make the required amount.  Prepare at the time of use.  A medium in which serum is removed from the Pericyte passage medium |
| C001.7 | Mix by inverting. Fill in the bottle with "Pericyte basal medium", "Date" and "Worker name". | Store at 4°C and use within 1month. |
| - MEMO | | |

※No: Inside the clean bench

**C002．Culture procedure for HBPC /ci37 Cell seeding from frozen cell**

- Date & Time： Y M D（ ） ： ～ ：
- Operator：（ ）
- Room Temperature： ℃

| No | Process | Note | |
| --- | --- | --- | --- |
| C002.1 | Preparation and confirmation of necessary items  〇Equipment：  Water bath, Clean bench, Centrifuge, Electric pipettor, Timer Clock, Dewar bottle for Liquid Nitrogen, Tweezers, Cell counter, Liquid nitrogen storage tank, Pipette, CO_2_ incubator（33℃, 5% CO_2_）  *Warm water bath at 37℃  〇Supplies：  Pipettes, Pipette Tips, 50mL tube, 15mL tube, Sterilized storage bottle, φ100mm dish (Collagen type I-coated), Hemocytometer  〇Reagents：  □Pericyte passage medium (Store at 4°C)  Basal Medium, 2%FBS, 1% Pericyte Growth Supplement, 1% Penicillin/streptomycin, 4µg/mL Blasticidin S  〇Cell：HBVC/ci37  Passage # , (1.0x10^6^ cells/vial)  Date , Lot | All equipment should be sterilized with 70% ethanol and placed in a clean bench.  Collagen type I coating（Refer to 001）  Use within 2weeks (Refer to C001） | |
| C002.2 | Pericyte passage medium to warm to the room temperature.  ： ～ ： |  | |
| C002.3 | Dispense the required amount (about 10mL/tube) of Pericyte passage medium into a conical tube. |  | |
| C002.4 | Write the "cell name", " Pericyte passage number", "worker name", and "date" on the lid of the Collagen type I-coated φ100 mm dish |  | |
| C002.5 | Remove the frozen cell stock from the liquid nitrogen cell storage tank and move it in a Dewar bottle. |  | |
| C002.6 | Take out the cryotube with tweezers, and warm it in a 37°C water bath while shaking. | Avoid getting water on the lid to prevent contamination.  Thaw frozen fluid to the extent that it moves away from the wall. | |
| C002.7 | The cryotube should be sterilized with 70% ethanol and placed in the clean bench. |  | |
| C002.8 | Transfer cells from cryotube to conical tube pre-dispensed with medium |  | |
| C002.9 | Rinse the cryotube with cell suspension and return to the conical tube. |  | |
| C002.10 | Centrifuge the conical tube at 120xg for 3min. |  | |
| C002.11 | Remove the supernatant with an ejector to the extent that the cells are not exposed, and loosen the cell pellet by tapping. |  | |
| C002.12 | Add 10mL of Pericyte passage medium along tube wall |  | |
| C002.13 | Slowly suspend by pipetting with a 10mL pipette. | Vortex strictly prohibited. | |
| C002.14 | Wash φ100mm dish (Collagen type I coated)（C002.4）with Pericyte passage medium twice and seed cells. | No need washing in the case of pre-corting products. | |
| C002.15 | Incubate cells at 33℃ with 5% CO_2._  ： ～ ： | 70-80% confluent for 2-3 days | |
| - MEMO | | |  |

※No: Inside the safety cabinet

**C003．Culture procedure for HBPC/ci37 Subculture**

- Date & Time： Y M D（ ） ： ～ ：
- Operator：（ ）
- Room Temperature： ℃

| No | Process | Note |
| --- | --- | --- |
| C003.1 | Preparation and confirmation of necessary items  〇Equipment：  Water bath, Clean bench, Centrifuge, Electric pipettor, Timer Clock, Pipette, CO_2_ incubator（33℃, 5% CO_2_）  〇Supplies：  Pipettes, Pipette Tips, 50mL tube, 15mL tube, Sterilized storage bottle, φ10mm dish/φ150 mm dish (Collagen type I-coated), Hemocytometer  〇Reagents：  □Pericyte passage medium (Store at 4°C)  Basal Medium, 2% FBS, 1%Pericyte Growth Supplement, 1% Penicillin/streptomycin, 4µg/mL Blasticidin S  □PBS(-)(Gibco, 14190-144)  □0.25% Trypsin-EDTA (1×) (Gibco, 25200-056)  Dilute to 0.1% Trypsin-EDTA with PBS (-).  □0.4% Trypan blue solution (SIGMA, T8154)  〇Cell：HBPC/ci37  Passage # , (1.0x10^6^ cells/vial)  Date , Lot | All equipment should be sterilized with 70% ethanol and placed in a clean bench  Collagen type I coating（refer to 001）,or use the pre-coating products (IWAKI, #4020-010）  Use within 2 weeks（Refer to C001）  Observe the expiration date of the medium. |
| C003.2 | Pericyte passage medium to warm to the room temperature.  ： ～ ： |  |
| C003.3 | Dilute 0.25% Trypsin-EDTA mL with mL PBS (-) to make 0.1% Trypsin-EDTA. |  |
| C003.4 | Remove from the 33°C 5% CO2 incubator and observe HBVPC/ci37. | Make sure that the cell confluency is 80% or more. |
| C003.5 | Remove the culture medium of HBPC/ci37 cells by suction. |  |
| C003.6 | Wash HBPC/ci37 cells with PBS (-).  PBS volume: φ100mm dish (5-10mL), φ150mm dish (20-30mL) |  |
| C003.7 | Remove PBS (-) by suction and add 0.1% Trypsin-EDTA (C003.3) to spread throughout the cells.  Trypsin volume: φ100 mm dish (2mL), φ150 mm dish (5mL) |  |
| C003.8 | Transfer the dish to a 33°C 5% CO_2_ incubator and warm for 3-4 minutes.  ： ～ ： |  |
| C003.9 | Tap the side of dish with the lid on, and confirm visually and microscopically that the cells have peeled off. |  |
| C003.10 | Add astrocyte passage medium. Adjust the amount of medium according to the dilution rate (see below). Volume: φ100mm dish (7-8mL), φ150mm dish (15-25mL) | Inactivate trypsin by FBS in medium |
| C003.11 | Slowly resuspend with an electric pipettor. | Vortex and intense suspension are strictly prohibited. |
| C003.12 | Collect the cell suspension in a conical tube.  Cell suspension（A ）mL |  |
| C003.13 | Take a portion of the suspension of C003.12 and the trypan blue solution by pipetting.  Cell suspension: Trypan blue solution = 1:1  … 2-fold dilution (*) |  |
| C003.14 | Add the suspension to the hemocytometer and count the cell number of four corners on the hemocytometer under a microscope.  B＝（ ＋ ＋ ＋ ）÷4×2（＊）  Cell density  ＝[B ]×10^4^ cells/mL  ＝[C ]×10^6^ cells/mL  total cell number  [C ]×10^6^ cells/mL× [A ] mL  ＝[D ]×10^6^ cells | Count cells without dyed with trypan blue as living cells. |
| C003.15 | Write the "cell name", "passage number", "worker name", and "date" on the lid of the Collagen type I-coated φ100mm dish  Medium Volume: φ100mm dish (5-10mL), φ150mm dish (20-30mL) | No need washing in the case of pre-coating products. |
| C003.16 | After removing the wash medium from the Dish, add an amount of astrocyte passage medium according to the dilution rate and re-seed the HBPC/ci37 cell suspension of C003.12 (Dilution rate: 1:2 to 1:4). | Cell density at 1×10^6^ cells/dish (100mm) or more.  Doubling time: 48～72hr. |
| C003.17 | Write the "cell name", "Pericyte passage number", "worker name", and "date" on the lid of the Collagen type I-coated φ100mm dish |  |
| C003.18 | Incubate cells at 33°C with 5% CO_2._  ： ～ ： | 70-80% confluent for 3-4 days. |
| - MEMO | | |

※No: Inside the clean bench

**C004．Culture procedure for HBPC/ci37 Cell stock**

- Date & Time： Y M D（ ） ： ～ ：
- Operator：（ ）
- Room Temperature： ℃

| No | Process | Note |
| --- | --- | --- |
| C004.1 | Preparation and confirmation of necessary items  〇Equipment：  Water bath, Clean bench, Centrifuge, Electric pipettor, Timer Clock, Pipette, CO_2_ incubator（33℃, 5% CO_2_）, Dewar bottle for Liquid Nitrogen, Tweezers, Cell counter, Liquid nitrogen storage tank  〇Supplies：  Pipettes, Pipette Tips, 50mL tube, 15mL tube, Sterilized storage bottle, φ100mm dish (Collagen type I-coated), Hemocytometer  〇Reagents：  □Pericyte passage medium(Store at 4°C)  Basal Medium, 2% FBS, 1% Pericyte Growth Supplement, 1% Penicillin/streptomycin, 4µg/mL Blasticidin S  □PBS(-)(Gibco, 14190-144)  □0.25% Trypsin-EDTA (1×) (Gibco, 25200-056)  Dilute to 0.1% Trypsin-EDTA with PBS (-).  □0.4% Trypan blue solution (SIGMA, T8154)  □Banbanker (Nippon genetics, CS-04-001)  〇Cell：HBPC/ci37  Passage # , (1.0x10^6^ cells/vial)  Date , Lot | All equipment should be sterilized with 70% ethanol and placed in a clean bench.  Cryotube:  Any tube that can be used for storage in liquid nitrogen  Use within 2 weeks (Refer to C001）. |
| C004.2 | Pericyte passage medium to warm to the room temperature.  ： ～ ： |  |
| C004.3 | Dilute 0.25% Trypsin-EDTA mL with mL PBS (-) to make 0.1% Trypsin-EDTA. |  |
| C004.4 | Remove from the 33°C 5% CO_2_ incubator and observe HBPC/ci37 cells. | Make sure that the cell confluency is 80% or more. |
| C004.5 | Remove the culture medium of HBPC/ci37 cells by suction. |  |
| C004.6 | Wash HBPC/ci35 cells with PBS (-).  PBS volume: φ100mm dish (5-10mL), φ150mm dish (20-30mL) |  |
| C004.7 | Remove PBS (-) by suction and add 0.1% Trypsin-EDTA (C003.3) to spread throughout the cells.  Trypsin volume: φ100mm dish (2mL), φ150mm dish (5mL) |  |
| C004.8 | Transfer the dish to a 33°C 5% CO_2_ incubator and warm for 3-4 minutes.  ： ～ ： |  |
| C004.9 | Tap the side of dish with the lid on, and confirm visually and microscopically that the cells have peeled off. |  |
| C004.10 | Add astrocyte passage medium.  Volume: φ100mm dish (8ml), 150mm dish (20ml) | Inactivate trypsin by FBS in medium |
| C004.11 | Slowly resuspend with an electric pipettor. |  |
| C004.12 | Collect the cell suspension in a conical tube.  Cell suspension（A ）mL |  |
| C004.13 | Take a portion of the suspension of C003.12 and mix the trypan blue solution by pipetting.  Cell suspension: Trypan blue solution = 1:1  … 2-fold dilution (*) |  |
| C004.14 | Add the suspension to the hemocytometer and count the cell number of four corners on the hemocytometer under a microscope.  B＝（ ＋ ＋ ＋ ）÷4×2（＊）  Cell density  ＝[B ]×10^4^ cells/mL  ＝[C ]×10^6^ cells/mL  total cell number  [C ]×10^6^ cells/mL× [A ] mL  ＝[D ]×10^6^ cells  Resuspend in a [D ] mL Banbanker. | Count cells without dyed with trypan blue as living cells.  Make stock  1×10^6^cells/tube/mL |
| C004.15 | Centrifuge the conical tube at 120xg for 3min. |  |
| C004.16 | Remove the supernatant by suction, add a [D ] mL Banbanker, and slowly suspend with an electric pipettor. |  |
| C004.17 | Place 1 mL / tube of Banbanker cell suspension (C004.16) in a cryotube and lid tightly. | The lid is sterilized by heating |
| C004.18 | Write the "cell name", "passage number", "Cell density” "worker name", and "date" on the tube.  Place in a cell freezing container and freeze at -80°C for at least half a day.  ： ～ ： |  |
| C004.19 | Transfer the frozen cell stock to a liquid nitrogen cell storage tank.  Date： / | After freezing at -80°C transfer as soon as possible. |
| - MEMO | | |

※No: Inside the clean bench

**D001．Culture procedure for HBMEC/ci18 Preparation for medium**

- Date & Time： Y M D（ ） ： ～ ：
- Operator：（ ）
- Room Temperature： ℃

| No | Process | Note |
| --- | --- | --- |
| D001.1 | Preparation and confirmation of necessary items  〇Equipment：  Clean bench, Electric pipettor, Pipette  〇Supplies：  Pipettes, Pipette Tips, 50mL tube, Sterilized storage bottle  〇Reagents：  　Endothelial cell basal Medium  -VascuLife VEGF complete kit  (LiFELINE CELL TECHNOLOGY（KURABO), LEC-LL0003)  -VascuLife basal medium (LEB-0002) (4℃）  475mL/bottle, pH 7.8±0.3  VascuLife VEGF Life Factors kit (LS-1020）(-20℃）  L-Glutamine (200 mM), VEGF (5µg/mL）,  hEGF (5µg/mL）, hFGF-b (5µg/mL）, hIGF-1 (15µg/mL）,  Ascorbic acid (50mg/mL) , Hydrocortisone Hemisuccinate (1mg/mL）, Heparin (750unit/mL) , FBS  　Penicillin/streptomycin（Gibco, 15140-122）  　Blasticidin S Hydrochloride（Wako, 029-18701）*  Stock solution: Prepared at 4 mg/mL (1000X) with dH_2_O. 0.22µm filter sterilization. Storage at 4°C. Use within 1month.  Date： | All equipment should be sterilized with 70% ethanol and placed in a clean bench.  Do not warm the medium, keep it below 33°C.  Use up the medium supplement by the expiration date. 　Supplement can be re-frozen up to once.  Blasticidin S: Avoid freeze/thaw cycles. |
| D001.2 | The following reagents are mixed to prepare the Endothelial cell basal medium.  VascuLife basal medium 190mL  L-Glutamine (200 mM) 10mL  VEGF (5µg/ml 0.2mL  hEGF (5µg/ml） 0.2mL  hFGF-b (5µg/ml） 0.2mL  hIGF-1 (15µg/ml） 0.2mL  Ascorbic acid (50mg/ml） 0.2mL  Hydrocortisone Hemisuccinate (1mg/ml） 0.2mL  Heparin (750unit/ml） 0.2mL  FBS 4mL  Penicillin/streptomycin 2mL  200mL  Date： | Final concentration:  10mM L-Glutamine  5ng/mL VEGF  5ng/mL hEGF  5ng/mL hFGF-b  15ng/mL hIGF-1  50µg/mL Ascorbic acid  1µg/mL Hydrocortisone  0.75unit/mL Heparin  2% (V/V) FBS  1% Pen/St |
| D001.3 | Move the bottle horizontally in a circular motion to stir. Fill in the bottle with " Endothelial cell basal medium", "Date" and "Worker name". | Avoid foaming when stirring.  Store at 4 ° C and use within 2 weeks. |
| D001.4 | Preparation of Endothelial cell passage medium  In the case of 1000-fold dilution,  Endothelial cell basal medium 50mL  Blasticidin S 4 mg / mL stock 50µL  Date： | Use up the medium supplement by the expiration date. 　Supplement can be re-frozen up to once.  To prevent Blasticidin from deactivating, small volume preparation at once are recommended. |
| D001.5 | Mix by inverting. Fill in " Endothelial cell passage medium", "Date" and "Worker name". | Store at 4 ° C and use within 2 weeks. |
| D001.6 | Preparation of Endothelial cell differentiation medium  VascuLife basal medium 47.5mL  L-Glutamine (200mM) 2.5mL  hFGF-b (5µg/mL) 0.05mL  hIGF-1 (15µg/mL) 0.05mL  Ascorbic acid (50mg/mL） 0.05mL  Hydrocortisone Hemisuccinate (1mg/mL) 0.05mL  Heparin (750unit/ml） 0.05mL  FBS (2% V/V） 1mL  Penicillin/streptomycin 0.5mL  50mL  Date： | Make the required amount.  Prepare at the time of use.  A medium in which VEGF and EGF is removed from the Endothelial cell passage medium |
| A002.7 | Mix by inverting. Fill in the bottle with " Endothelial cell basal medium", "Date" and "Worker name". | Store at 4 ° C and use within 2 weeks. |
| - MEMO | | |

※No: Inside the clean bench

**D002．Culture procedure for HBMEC/ci18 Cell seeding from frozen cell**

- Date & Time： Y M D（ ） ： ～ ：
- Operator：（ ）
- Room Temperature： ℃

| No | Process | Note |
| --- | --- | --- |
| D002.1 | Preparation and confirmation of necessary items  〇Equipment：  Water bath, Clean bench, Centrifuge, Electric pipettor, Timer Clock, Dewar bottle for Liquid Nitrogen, Tweezers, Cell counter, Liquid nitrogen storage tank, Pipette, CO_2_ incubator（33℃, 5% CO_2_）  *Warm water bath at 37℃  〇Supplies：  Pipettes, Pipette Tips, 50mL tube, 15mL tube, Sterilized storage bottle, φ100mm dish (Collagen type I-coated), Hemocytometer  〇Reagents：  □Endothelial cell passage medium (Store at 4°C)  Basal medium, 10 mM L- Glutamine, 5ng/mL VEGF, 5ng/mL hEGF, 5ng/mL hFGF-b, 15ng/mL hIGF-1, 50µg/mL Ascorbic acid, 1µg/mL Hydrocortisone Hemisuccinate, 0.75unit/mL Heparin, 2% FBS, 1% Penicillin/streptomycin, 4µg/mL Blasticidin S  〇Cell： HBMEC/ci18  P Passage # , (1.0x10^6^ cells/vial)  Date , Lot | All equipment should be sterilized with 70% ethanol and placed in a clean bench.  Collagen type I coating（Refer to 001）  Use within 2 weeks (Refer to D001). |
| D002.2 | Endothelial passage medium to warm to the room temperature.  ： ～ ： |  |
| D002.3 | Dispense the required amount (about 10mL/tube) of Endothelial cell passage medium into a conical tube. |  |
| D002.4 | Write the "cell name", "passage number", "worker name", and "date" on the lid of the Collagen type I-coated φ100 mm dish |  |
| D002.5 | Remove the frozen cell stock from the liquid nitrogen cell storage tank and move it in a Dewar bottle. |  |
| D002.6 | Take out the cryotube with tweezers, and warm it in a 37°C water bath while shaking. | Avoid getting water on the lid to prevent contamination.  Thaw frozen fluid to the extent that it moves away from the wall. |
| D002.7 | Should be disinfected with 70% ethanol and placed in a safety cabinet. |  |
| D002.8 | Transfer cells from cryotube to conical tube pre-dispensed with medium |  |
| D002.9 | Rinse the cryotube with cell suspension and return to the conical tube. |  |
| D002.10 | Centrifuge the conical tube at 120xg for 3min. |  |
| D002.11 | Remove the supernatant with an ejector to the extent that the cells are not exposed, and loosen the cell pellet by tapping. |  |
| D002.12 | Add 10 mL of Endothelial cell passage medium along tube wall |  |
| D002.13 | Slowly suspend by pipetting with a 10mL pipette. | Vortex strictly prohibited. |
| D002.14 | Wash φ100mm dish (Collagen type I coated)（D002.4）with Endothelial cell passage medium twice and seed cells. | No need washing in the case of pre-corting products. |
| D002.16 | Incubate cells at 33℃ with 5% CO_2._  ： ～ ： | 70-80% confluent for 3-4 days |
| - MEMO | | |

※No: Inside the clean bench

**D003．Culture procedure for HBMEC/ci18_Subculture**

- Date & Time： Y M D（ ） ： ～ ：
- Operator：（ ）
- Room Temperature： ℃

| No | Process | Note |
| --- | --- | --- |
| D003.1 | Preparation and confirmation of necessary items  〇Equipment：  Clean bench, Electric pipettor, Pipette  〇Supplies：  Pipettes, Pipette Tips, 50mL tube, Sterilized storage bottle  〇Reagents：  □Endothelial cell passage medium (Store at 4°C)  Basal medium, 10 mM L- Glutamine, 5ng/mL VEGF, 5ng/mL hEGF, 5ng/mL hFGF-b, 15ng/mL hIGF-1, 50µg/mL Ascorbic acid, 1µg/mL Hydrocortisone Hemisuccinate, 0.75unit/mL Heparin, 2% FBS, 1% Penicillin/streptomycin, 4µg/mL Blasticidin S  PBS (-)(Gibco, 14190-144)  □0.25% Trypsin-EDTA (1×) (Gibco, 25200-056)  Dilute to 0.1% Trypsin-EDTA with PBS (-). (Prepared before use)  □0.4% Trypan blue solution (SIGMA, T8154)  〇Cell: HBMEC/ci18  Passage # , (1.0x10^6^ cells/vial)  Date , Lot | All equipment should be sterilized with 70% ethanol and placed in a clean bench.  Collagen type I coating（Refer to 001）  Or use the pre-coating products (IWAKI, #4020-010）  Use within 2 weeks（Refer to A002）.  Observe the expiration date of the medium. |
| D003.2 | Endothelial cell passage medium to warm to the room temperature.  ： ～ ： |  |
| D003.3 | Dilute 0.25% Trypsin-EDTA mL with mL PBS (-) to make 0.1% Trypsin-EDTA. | Dilute with 1.5times of PBS (-) against the amount of 0.25% Trypsin. |
| D003.4 | Remove from the 33°C 5% CO2 incubator and observe HBMEC/ci18cells. | Make sure that the cell confluency is 80% or more. |
| D003.5 | Remove the culture medium of Endothelial cell cells by suction. |  |
| D003.6 | Wash Endothelial cell cells with PBS (-).  PBS volume:φ100 m dish (5-10mL), φ150 m dish (20-30mL) |  |
| D003.7 | Remove PBS (-) by suction and add 0.1% Trypsin-EDTA (D003.3) to spread throughout the cells.  Trypsin volume: φ100 mm dish (2mL), φ150 mm dish 5mL) |  |
| D003.8 | Transfer the dish to a 33°C 5% CO_2_ incubator and warm for 3-4 minutes.  ： ～ ： |  |
| D003.9 | Tap the side of dish with the lid on, and confirm visually and microscopically that the cells have peeled off. |  |
| D003.10 | Add Endothelial cell passage medium. Adjust the amount of medium according to the dilution rate (see below). Volume: φ100mm dish (7-8mL), φ150mm dish (15-25mL) | Inactivate trypsin by FBS in medium |
| D003.11 | Slowly resuspend with an electric pipettor. | Vortex and intense suspension are strictly prohibited. |
| D003.12 | Collect the cell suspension in a conical tube.  Cell suspension（A ）mL |  |
| D003.13 | Take a portion of the suspension of D003.12 and mix with the trypan blue solution by pipetting.  Cell suspension: Trypan blue solution = 1:1  … 2-fold dilution (*) |  |
| D003.14 | Add the suspension to the hemocytometer and count the cell number of four corners on the hemocytometer under a microscope.  B＝（ ＋ ＋ ＋ ）÷4×2（＊）  Cell density  ＝[B ]×10^4^ cells/mL  ＝[C ]×10^6^ cells/mL  total cell number  [C ]×10^6^ cells/mL× [A ] mL  ＝[D ]×10^6^ cells | Count cells without dyed with trypan blue as living cells. |
| D003.15 | Write the "cell name", "passage number", "worker name", and "date" on the lid of the Collagen type I-coated φ100 mm dish  Medium Volume: φ100mm dish (5-10mL), φ150mm dish (20-30mL) | No need washing in the case of pre-corting products. |
| D003.16 | After removing the wash medium from the Dish, add an amount of astrocyte passage medium according to the dilution rate and re-seed the HBMEC/ci18 cell suspension of D003.12 (Dilution rate: 1:2).  Total medium volume per dish: φ100mm (10mL), φ150mm (30mL)  Record the dilution rate and the number of dishes. | Cell density at 1×10^6^ cells/dish (100mm) or more.  Doubling time:72～96hr |
| D003.17 | Write the "cell name", "Endothelial cell passage number", "worker name", and "date" on the lid of the Collagen type I-coated φ100mm dish |  |
| D003.18 | Incubate cells at 33℃ with 5% CO_2._  ： ～ ： | 70-80% confluent for 3-4 days. |
| - MEMO | | |

※No: Inside the clean bench

**D004．Culture procedure for HBMEC/ci18_Cell stock**

- Date & Time： Y M D（ ） ： ～ ：
- Operator：（ ）
- Room Temperature： ℃

| No | Process | Note |
| --- | --- | --- |
| D004.1 | Preparation and confirmation of necessary items  〇Equipment：  Water bath, Clean bench, Centrifuge, Electric pipettor, Timer Clock, Pipette, CO_2_ incubator（33℃, 5% CO_2）_, Dewar bottle for Liquid Nitrogen, Tweezers, Cell counter, Liquid nitrogen storage tank  〇Supplies：  Pipettes, Pipette Tips, 50mL tube, 15mL tube, Sterilized storage bottle, φ100 mm dish (Collagen type I-coated), Hemocytometer  Cryotube:  〇Reagents：  □Endothelial cell passage medium (Store at 4°C)  Basal medium, 10 mM L- Glutamine, 5ng/mL VEGF, 5ng/mL hEGF, 5ng/mL hFGF-b, 15ng/mL hIGF-1, 50µg/mL Ascorbic acid, 1µg/mL Hydrocortisone Hemisuccinate, 0.75unit/mL Heparin, 2% FBS, 1% Penicillin/streptomycin, 4µg/mL Blasticidin S    □PBS (-)(Gibco, 14190-144)  □0.25% Trypsin-EDTA (1×) (Gibco, 25200-056)  Dilute to 0.1% Trypsin-EDTA with PBS (-)  □0.4% Trypan blue solution (SIGMA, T8154)  □Banbanker (Nippon genetics, CS-04-001)  〇Cell： HBMEC/ci18  P Passage # , (1.0x10^6^ cells/vial)  Date , Lot | All equipment should be sterilized with 70% ethanol and placed in a clean bench  Cryotube:  Any tube that can be used for storage in liquid nitrogen  Use within 2weeks (Refer to A002). |
| D004.2 | Endothelial cell passage medium to warm to the room temperature.  ： ～ ： |  |
| D004.3 | Dilute 0.25% Trypsin-EDTA mL with mL PBS (-) to make 0.1% Trypsin-EDTA. | Dilute with 1.5 times of PBS (-) against the amount of 0.25% Trypsin. |
| D004.4 | Remove the culture medium of HBMEC/ci18 cells by suction. |  |
| D004.5 | Wash HBMEC/ci18 cells with PBS (-).  PBS volume: φ100mm dish (5-10mL), φ150mm dish (20-30mL) |  |
| D004.6 | Remove PBS (-) by suction and add 0.1% Trypsin-EDTA (D003.3) to spread throughout the cells.  Trypsin volume: φ100mm dish (2mL), φ150mm dish (5mL) |  |
| D004.7 | Transfer the dish to a 33°C 5% CO2 incubator and warm for 3-4 minutes.  ： ～ ： |  |
| D004.8 | Remove the culture medium of HBMEC/ci18 cells by suction. |  |
| D004.9 | Tap the side of dish with the lid on, and confirm visually and microscopically that the cells have peeled off. |  |
| D004.10 | Add Endothelial cell passage medium.  Volume: φ100 mm dish (8ml), 150 mm dish (20ml) | Inactivate trypsin by FBS in medium |
| D004.11 | Slowly resuspend with an electric pipettor. | Vortex and intense suspension are strictly prohibited. |
| D004.12 | Collect the cell suspension in a conical tube.  Cell suspension（A ）mL |  |
| D004.13 | Take a portion of the suspension of D003.12 and mix with the trypan blue solution by pipetting.  Cell suspension: Trypan blue solution = 1:1  … 2-fold dilution (*) |  |
| D004.14 | Add the suspension to the hemocytometer and count the cell number of four corners on the hemocytometer under a microscope.  B＝（ ＋ ＋ ＋ ）÷4×2（＊）  Cell density  ＝[B ]×10^4^ cells/mL  ＝[C ]×10^6^ cells/mL  total cell number  [C ]×10^6^ cells/mL× [A ] mL  ＝[D ]×10^6^ cells  Resuspend in a [D ] mL Banbanker. | Count cells without dyed with trypan blue as living cells.  Make the stock 1×10^6^cells/tube/mL |
| D004.15 | Centrifuge the conical tube at 120xg for 3min. |  |
| D004.16 | Remove the supernatant by suction, add a [D ] mL Banbanker, and slowly suspend with an electric pipettor. |  |
| D004.17 | Place 1 mL/tube of Banbanker cell suspension (D004.16) in a cryotube and lid tightly. | The lid is sterilized by heating |
| D004.18 | Write the "cell name", "passage number", "Cell density” "worker name", and "date" on the tube.  Place in a cell freezing container and freeze at -80°C for at least half a day.  ： ～ ： |  |
| D004.19 | Transfer the frozen cell stock to a liquid nitrogen cell storage tank.  Date： / | After freezing at -80°C transfer as soon as possible. |
| - MEMO | | |
|  |  |  |

※No: Inside the clean bench

**E001．Human immortalized cell-based BBB tricellular model Plate coating for HASTR/ci35**

- Date & Time： Y M D（ ） ： ～ ：
- Operator：（ ）
- Room Temperature： ℃

| No | Process | note |
| --- | --- | --- |
| E001.1 | Preparation and confirmation of necessary items  〇Equipment：  Clean bench, Electric pipettor, Pipette  〇Supplies：  Pipettes, Pipette Tips, 50mL tube  〇Reagents：  □Cellmatrix Type I-C 3mg/mL 100mL (Nitta gelatin)  □Media for each cell  □Dilute hydrochloric acid 1mM, 0.001N  After measuring 100µL HCL（5mol/L, Wako, 081-05435）with ultrapure water to make 500mL, autoclave sterilization.  〇Culture dishes：  　24well plate (Cell culture multiwell plate 24well plate, Greiner-bio-one, 662160） | All equipment should be sterilized with 70% ethanol and placed in a clean bench.  Dilute hydrochloric acid: Filter sterilization of φ0.2 µm pore is also possible. |
| E001.2 | Prepare the required amount of collagen solution.  500µl× wells ＝A ml  The amount of Cellmatrix Type I-C solution  A ml /10＝B ml  The amount of 1mM HCL  A ml- B ml ＝C ml |  |
| E001.3 | Add 500µll collagen solution to the each well of culture plate. |  |
| E001.4 | Let stand in the clean bench for 30minutes  ： ～ ： |  |
| E001.5 | Aspirate and remove the collagen solution. |  |
| E001.6 | Open the lid and let it dry. (1 to 2hours)  ： ～ ：  UV irradiation for 15minutes  ： ～ ： |  |
| E001.7 | After drying, fill in the "date" and "worker name", wrap it, and store it in the clean bench. | Can be stored for 1 to 2 months.  On the day of cell seeding, wash twice with culture medium before use, and then add the cell suspension. |
| - Memo | | |

※No: Inside the clean bench

**E002．Human immortalized cell-based BBB tricellular model Coating of transwell for HBMEC/ci18 and HBPC/ci37**

- Date & Time： Y M D（ ） ： ～ ：
- Operator：（ ）
- Room Temperature： ℃

| No | Process | Note |
| --- | --- | --- |
| E002.1 | Preparation and confirmation of necessary items  〇Equipment：  Clean bench, Electric pipettor, Pipette  〇Supplies：  Pipettes, Pipette Tips, 50mL tube  〇Reagents：  □Cellmatrix Type IV 3mg/mL 100mL (Nitta gelatin)  □Media for each cell  □Dilute hydrochloric acid 1mM, 0.001N  After measuring 100µL HCL（5mol/L, Wako, 081-05435）with ultrapure water to make 500mL, autoclave sterilization.  on.  〇Culture dishes：  □24well plate (Cell culture multi well plate 24well plate, Greiner-bio-one, 662160)  □Transwell Millicell cell culture insert 24well hanging Inserts 0.4µm PET, Millipore, MCHT24H48 | All equipment should be sterilized with 70% ethanol and placed in a clean bench. |
| E002.2 | Prepare the required amount of ECM solution.  500µl× wells ＝A ml  The amount of Cellmatrix Type Ⅳ solution  A ml/30＝ B ml  The amount of Fibronectin  A ml/20= C ml  The amount of 1mM HCL  A ml- B+C ml ＝D ml |  |
| E002.3 | Put the culture inserts into the well of the 24well plate. |  |
| E002.4 | Add 150µl of ECM solution to each transwell and 350µl of ECM solution to each well of culture plate. |  |
| E002.5 | Let stand in the clean bench for 30minutes  ： ～ ： |  |
| E002.6 | Aspirate and remove the ECM solution. |  |
| E002.7 | Open the lid and let it dry for 20minutes  ： ～ ： |  |
| E002.8 | Add 150µl of PBS (-) to transwell and 350µl PBS (-) to well of culture plate.  The amount of PBS (-)  A ml |  |
| E002.9 | Aspirate and remove PBS (-). |  |
| E002.10 | Open the lid and let it dry. (1 to 2 hours)  ： ～ ：  UV irradiation for 15minutes  ： ～ ： |  |
| E002.11 | After drying, fill in the "date" and "worker name", wrap it, and store it in the clean bench. | Can be stored for 1month |
| - MEMO | | |

※No: Inside the clean bench

**E003. Human immortalized cell-based BBB tricellular model HBPC/ci37 Seeding and Induction of differentiation**

- Date & Time： Y M D（ ） ： ～ ：
- Operator：（ ）
- Room Temperature： ℃

| No | Process | note |
| --- | --- | --- |
| E003.1 | Preparation and confirmation of necessary items  〇Equipment：  Clean bench, Electric pipettor, Pipette, Water bath, Timer Clock, Tweezers, Cell counter, CO_2_ incubator（33℃, 5% CO_2_）  〇Supplies：  Pipettes, Pipette Tips, 50mL tube, 15mL tube, Sterilized storage bottle, φ100mm dish, Transwell (Collagen type IV, FN-coated), Hemocytometer  〇Reagents：  □Pericyte passage medium (Store at 4°C)  Basal Medium, 2% FBS, 1% Pericyte Growth Supplement, 1% Penicillin/streptomycin,4µg/ml Blasticidin S  □Pericyte differentiation medium (Store at 4°C)  Basal Medium, 1% Pericyte Growth Supplement, 1% Penicillin/streptomycin  □PBS(-) (Gibco, 14190-144)  □0.25% Trypsin-EDTA (1×) (Gibco, 25200-056)  Dilute to 0.1% Trypsin-EDTA with PBS (-).  □0.4% Trypan blue solution (SIGMA, T8154)  □Sterilized water  〇Culture dish：  □φ100mm dish（Corning, 430167）  □24well plate  (Cell culture multiwell plate 24well plate, Greiner-bio-one, 662160）  □Transwell (Collagen type IV, FN-coated)  （Millicell cell culture insert 24well hanging Inserts 0.4µm PET, Millipore, MCHT24H48）  〇Cell：HBPC/ci37  Passage #  Date | All equipment should be sterilized with 70% ethanol and placed in a clean bench  Use medium within 2weeks (Refer to B002).  Coating (Refer to E002) |
| E003.2 | **HBPC/ci37 seeding 2 days before the start of coculture**  Pericyte passage medium, and 0.25% Trypsin-EDTA to warm to the room temperature.  ： ～ ：  Dilute 0.25% Trypsin-EDTA mL with mL PBS (-) to make 0.1% Trypsin-EDTA. |  |
| E003.3 | Place Transwell (Collagen type IV, FN-coated) on a φ100mm dish with the PVDF membrane side facing up. Fill with 5ml of sterile water avoiding dry. |  |
| E003.4 | Remove from the 33°C 5% CO_2_ incubator and observe HBPC/ci37 cells. | Make sure that the cell confluency is 80% or more. |
| E003.5 | Trypsinize HBPC/ci37 cells to obtain cell suspension | Refer to B004.3～11 |
| E003.6 | Collect the cell suspension in a conical tube.  Cell suspension（A ）mL |  |
| E003.7 | Take a portion of the suspension of E003.6 and mix with the trypan blue solution by pipetting.  Cell suspension: Trypan blue solution = 1:1  … 2-fold dilution (*) |  |
| E003.8 | Add the suspension to the hemocytometer and count the cell number of four coners on the hemocytometer under a microscope.  B＝（ ＋ ＋ ＋ ）÷4×2（*）  Cell density  ＝[B ]×10^4^ cells/mL  ＝[C ]×10^6^ cells/mL  total cell number  [C ]×10^6^ cells/mL× [A ] mL  ＝[D ]×10^6^ cells | Count cells without dyed with trypan blue as living cells. |
| E003.9 | Adjust cell suspension to 1 x 10^5^ cells/ml |  |
| E003.10 | Overlay 100µl of cell suspension (1 x 10^4^ cells) on PVDF membrane |  |
| E003.11 | Cover the φ100mm dish avoiding dry and let stand at room temperature for 45minutes. |  |
| E003.12 | Add 500µl/well of pericyte subculture medium to a 24well plate. |  |
| E003.13 | After 45minutes, set the Transwell seeded with cells on the plate. |  |
| E003.14 | Transfer to a 33°C incubator and incubate for 24hours. |  |
| E003.15 | **The next day (one day before the start of co-culture), induce differentiation of HBPC/ci37.**  Return the differentiation medium to room temperature |  |
| E003.16 | Add 500µl/well of pericyte differentiation medium to a new 24well plate. |  |
| E003.17 | Wash the Transwell seeded with HBPC/ci37 cell with 500µl PBS (-) (twice) to remove serum. |  |
| E003.18 | Transfer the Transwell seeded with HBPC/ci37 cells to new plate. |  |
| E003.19 | Transfer to a 37°C incubator and incubate for 24hours. |  |
| - MEMO | | |

※No: Inside the clean bench

**E004．Human immortalized cell-based BBB tricellular model HASTR/ci35 Seeding and Induction of differentiation**

- Date & Time： Y M D（ ） ： ～ ：
- Operator：（ ）
- Room Temperature： ℃

| No | Process | note |
| --- | --- | --- |
| E004.1 | Preparation and confirmation of necessary items  〇Equipment：  Clean bench, Electric pipettor, Pipette, Water bath, Centrifuge, Electric pipettor, Timer Clock, Cell counter, CO_2_ incubator（33℃, 5% CO_2_）  〇Supplies：  Pipettes, Pipette Tips, 50mL tube, 15mL tube, Sterilized storage bottle, φ100 mm dish, Transwell (Collagen type IV, FN-coated), Hemocytometer  〇Reagents：  □Astrocyte passage medium (Store at 4°C)  （DMEM+Glutamax, 1% N2 supplement, 10% FBS,  1% Penicillin/streptomycin, 4µg/ml Blasticidine S）  □Astrocyte differentiation medium (Store at 4°C)  （DMEM+Glutamax, 1% N2 supplement,  1% Penicillin/streptomycin）  □cAMP  100mM stock, (Store at -20℃)  fnal concentration: 1mM  □PBS(-)(Gibco, 14190-144)  □0.25% Trypsin-EDTA (1X) (Gibco、25200-056)  Dilute to 0.1% Trypsin-EDTA with PBS (-)  □0.4% Trypan blue solution (SIGMA, T8154)  〇Culture dish：  □24well plate (Collagen type I-coated）  〇Cell：HASTR/ci35  Passage #  Date | All equipment should be sterilized with 70% ethanol and placed in a clean bench  Corting (refer to E001) |
| E004.2 | **HASTR/ci35 seeding 2 days before the start of coculture**  Pericyte passage medium, and 0.25% Trypsin-EDTA to warm to the room temperature.  ： ～ ：  Dilute 0.25% Trypsin-EDTA mL with mL PBS (-) to make 0.1% Trypsin-EDTA. |  |
| E004.3 | Remove from the 33°C 5% CO_2_ incubator and observe HASTR/ci35 | Make sure that the cell confluency is 80% or more. |
| E004.4 | Trypsinize HASTR/ci35 cells to obtain cell suspension | Refer to A004.3～11 |
| E004.5 | Collect the cell suspension in a conical tube.  Cell suspension（A ）mL |  |
| E004.6 | Take a portion of the suspension of E003.6 and mix with the trypan blue solution by pipetting.  Cell suspension: Trypan blue solution = 1:1  … 2-fold dilution (*) |  |
| E004.7 | Add the suspension to the hemocytometer and count the cell number of four corners on the hemocytometer under a microscope.  B＝（ ＋ ＋ ＋ ）÷4×2（*）  Cell density  ＝[B ]×10^4^ cells/mL  ＝[C ]×10^6^ cells/mL  total cell number  [C ]×10^6^ cells/mL× [A ] mL  ＝[D ]×10^6^ cells |  |
| E004.8 | Adjust cell suspension to 1 x 10^5^ cells/ml |  |
| E004.9 | Seed 500 µl of cell suspension on a 24well plate (Collagen type I-coated) (5x 10^4^ cells/well) |  |
| E004.10 | Transfer to a 33°C incubator and incubate for 24hours. |  |
| E004.11 | **The next day (one day before the start of co-culture), induce differentiation of HASTR/ci35.**  Return the differentiation medium to room temperature  ・Amount of medium  well×500ml =D ml  ・cAMP (diluent rate:1/100)  D＿＿＿＿＿＿ml×1/100＝E＿＿＿＿＿＿＿＿＿ml |  |
| E004.12 | Wash the well seeded with HASTR/ci35 cell with 500µl PBS (-) (twice) to remove serum. |  |
| E004.13 | Add 500µl/well of Astrocyte differentiation medium |  |
| E004.14 | Transfer to a 37°C incubator and incubate for 24hours. |  |
| - MEMO | | |

※No: Inside the clean bench

**E005．Human immortalized cell-based BBB tricellular model HBMEC/ci18 Seeding and Coculture**

- Date & Time： Y M D（ ） ： ～ ：
- Operator：（ ）
- Room Temperature： ℃

| No | Process | note |
| --- | --- | --- |
| E005.1 | Preparation and confirmation of necessary items  〇Equipment：  Clean bench, Electric pipettor, Pipette  Water bath, Safety cabinet, Centrifuge, Electric pipettor, Timer Clock, Cell counter, CO_2_ incubator（33℃, 5% CO_2_）  〇Supplies：  Pipettes, Pipette Tips, 50mL tube, 15mL tube, Sterilized storage bottle, φ100 mm dish, Transwell (Collagen type IV, FN-coated), Hemocytometer  〇Reagents：  □ Endothelial cell passage medium (Store at 4 °C)  Basal medium, 10mML-Glutamine, 5ng/ml VEGF, 5ng/ml hEGF, 5ng/ml hFGF-b, 15ng/ml hIGF-1, 50µg/ml Ascorbic acid, 1µg/ml Hydrocortisone Hemisuccinate, 0.75units/ml Heparin, 2% FBS, 1% Penicillin/streptomycin  □Endothelial cell differentiation medium (Store at 4 °C)  Basal medium, 10mM L- Glutamine, 5ng/ml hFGF-b, 15ng/ml hIGF-1, 50µg/ml Ascorbic acid, 1µg/ml Hydrocortisone Hemisuccinate, 0.75units/ml Heparin, 2% FBS, 1% Penicillin/streptomycin  □Brain side medium  ・Neurobasal medium (-) L-Glutamine (Gibco, 21103-049)  ・N2 supplement（Gibco, 17502-048）（100 X）  ・200mM Glutamate（Gibco, 25030-149）（100 X）  □PBS(-)(Gibco, 14190-144)  □0.25% Trypsin-EDTA (1X) (Gibco, 25200-056)  Dilute to 0.1% Trypsin-EDTA with PBS (-).  □0.4% Trypan blue solution (SIGMA, T8154)  〇Cell：HBMEC/ci185  Passage #  Date  Differentiated HBVPC/ci37  Differentiated HASTR/ci35 | All equipment should be sterilized with 70% ethanol and placed in a clean bench.  Use within 2 weeks.  Preparation Medium: Refer to C002.  Seeding and Induction: Refer to E003, E004 |
| E005.2 | Medium, and 0.25% Trypsin-EDTA to warm to the room temperature.  ： ～ ：  Dilute 0.25% Trypsin-EDTA mL with mL PBS (-) to make 0.1% Trypsin-EDTA. |  |
| E005.3 | Remove from the 33°C 5% CO_2_ incubator and observe HBMEC/ci18 | Make sure that the cell confluency is 80% or more. |
| E005.4 | Trypsinize HBMEC/ci18 cells to obtain cell suspension | Refer to C004.3～11 |
| E005.5 | Collect the cell suspension in a conical tube.  Cell suspension（A ）mL |  |
| E005.6 | Take a portion of the suspension of E003.6 and mix with the trypan blue solution by pipetting.  Cell suspension: Trypan blue solution = 1:1  … 2-fold dilution (*) |  |
| E005.7 | Add the suspension to the hemocytometer and count the cell number of four corners on the hemocytometer under a microscope.  B＝（ ＋ ＋ ＋ ）÷4×2（＊）  Cell density  ＝[B ]×10^4^ cells/mL  ＝[C ]×10^6^ cells/mL  total cell number  [C ]×10^6^ cells/mL× [A ] mL  ＝[D ]×10^6^ cells |  |
| E005.8 | Centrifuge the conical tube at 120xg for 3min. |  |
| E005.9 | Remove the supernatant by suction. |  |
| E005.10 | Add Endothelial cell differentiation medium and adjust cell suspension to 4 x 10^5^ cells/ml |  |
| E005.11 | Seed 250µl of cell suspension on a transwell with differentiated HBPC/ci37    (1×10^5^ cells/insert) |  |
| E005.12 | Remove HASTR/ci35 differentiation medium by suction from 24well plate that cultured Astrocyte., and add 1mL Brain side medium to the well. |  |
| E005.13 | Set the Transwell seeded with HBMEC/ci18 on a 24well plate of differentiated HASTR/ci35 cell culture. |  |
| E005.14 | Transfer to a 33°C incubator and incubate for 24hours. |  |
| E005.15 | After 24hours, Measurement TEER and use for analysis. |  |
| - MEMO | | |

※No: Inside the Clean bench

**F001．Human immortalized cell-based BBB tricellular model 　TEER Measurement**

- Date & Time： Y M D（ ） ： ～ ：
- Operator：（ ）
- Room Temperature： ℃

| No | Process | note |
| --- | --- | --- |
| F001.1 | Preparation and confirmation of necessary items  〇Equipment：  Clean bench, Electric pipettor, Pipette  Voltorhmmeter EVOM2, Chopstick Electrode STX2  〇Supplies：  Pipettes, Pipette Tips, 15mL tube  〇Reagents：  □ 70% ethanol  □ Brain side medium  ・Neurobasal medium (-) L-Glutamine (Gibco, 21103-049)  ・N2 supplement（Gibco, 17502-048）（100 X）  ・200mM Glutamate（Gibco, 25030-149）（100 X）  〇Cell：  Human immortalized cell-based BBB tricellular model (Coculture Day 1) | All equipment should be sterilized with 70% ethanol and placed in a clean bench. |
| F001.2 | Dispense 5 ml of 70% ethanol into a 15 ml tube  Immerse the electrode STX2 (3-5 minutes) and sterilize. |  |
| F001.3 | Set the voltohmmeter EVOM2, set the function mode to the Ohms position, and measure the test resistance.  __________Ω |  |
| F001.4 | Dispense 5 ml of Brain side medium into a 15 ml tube and immerse the electrode STX2. |  |
| F001.5 | Measure the resistances value from blank insert (no cells) by EVOM2 voltorhmmeter with chopstick Electrode STX2.  A＿＿＿＿＿＿Ω | ・Measure so that the insertion angle and depth of the electrodes in the insert are the same for all samples.  ・Wash the electrodes with detergent, in the case blank value is 210 Ω㎠ or more, and not stable. |
| F001.6 | Measure the resistances value from each BBB model insert by EVOM^2^ voltorhmmeter with STX2.  B＿＿＿＿＿＿Ω | ・Measure so that the insertion angle and depth of the electrodes in the insert are the same for all samples. |
| F001.7 | The net resistance value was calculated by subtracting the measured resistance value of blank from the measured resistance value of the BBB model.  TEER(Ω㎠)  = the net resistance value(B-A) ＿＿＿＿＿＿　(Ω）× 0.33 (surface area) (cm^2^) | ・The BBB models with a TEER value of over 10Ω㎠are used for permeability and bidirectional Transport assay |
| F001.8 | Wash STX2 with distilled water and dry. | ・Wash the electrodes with detergent, in the case it is very dirty. |
| - MEMO | | |

※No: Inside the Clean bench

**F002．Human immortalized cell-based BBB tricellular model Permeability and bidirectional Transport assay**

- Date & Time： Y M D（ ） ： ～ ：
- Operator：（ ）
- Room Temperature： ℃

| No | Process | note |
| --- | --- | --- |
| F002.1 | Preparation and confirmation of necessary items  〇Equipment：  Clean bench, Electric pipettor, Pipette  Safety cabinet, Centrifuge, Timer Clock, CO_2_ incubator（33℃/ 37℃, 5% CO_2_）,　Shaker, Vortex  〇Supplies：  Pipettes, Pipette Tips, 50mL tube, 24well plate, 96well plate, plate sheal  〇Reagents：  □D-PBSH  10mM HEPES, 25mM D-Glucose, D-PBS  □Substrate | ・All equipment should be sterilized with 70% ethanol and placed in a clean bench.  * 10 X D-PBS (Sigme, D1283-500ML) |
| F002.2 | Prepare the substrate solution and D-PBSH  ・Substrate solution  Dilute each substrate with D-PBSH to final concentration  Volume :（200µl for A to B assay , 900µl for B to A assay）  ×number of sample ＿＿＿＿  ＝　＿＿＿＿＿＿＿＿＿ml  ・D-PBSH  Stock as 5 X D-PBSH and dilute with dH_2_O before use.  Volume :（（900µl for wash + 1.1ml for assay) × sampling point number ＿＿＿＿＿ ）× number of sample ＿＿＿＿  ＝＿＿＿＿＿＿＿＿＿ml  After preparation, warm to 37 ℃ | ・Preparate for the culture inserts to be used as blank, at least one. Sample number includes the blank inserts.  ・Sampling points number  If the sample collection is at 15 minutes, 30 minutes, 45 minutes, 60 minutes, number is 4. |
| F002.3 | Prepare a 24-well plate**【**Wash plate**】**for washing the culture insert inoculated with cells before the test.  Add 900µl/well D-PBSH and warm to 37 ℃. |  |
| F002.4 | To perform a permeability assay from the blood vessel side to the brain side (A to B), prepare for 24 well plate**【**A to B assay plate**】**, the culture inserts to be used as blank.  Add 900µl/well D-PBSH in 24 well plate and warm to 37 ℃.  D-PBSH required volume (mL)  __________ × 900µl = ＿＿＿＿＿＿＿＿ml |  |
| F002.5 | **【A to B assay】**  Take the BBB model from the CO_2_ incubator (33 ℃, 5% CO_2_) to the clean bench.  Wash the BBB model to the【wash plate】before the assay. |  |
| F002.6 | Lift the BBB model inserts with tweezers, remove the medium from the insert by suction, and then return them to【Wash plate】. | ・Performed for each insert |
| F002.7 | Add 200 µl of substrate solution into the BBB model insert and transfer from【Wash plate】to【A to B assay plate】.  Start counting up the timer and record the time on a recording sheet.  Repeat for the remaining models and blank inserts. | ・Substrate solution should be vortexed well (more than 30 seconds)　before addition |
| F002.8 | Place【A to B assay plate】on a shaker in a CO_2_ incubator (37 ℃, 5% CO_2_) (50 rpm) |  |
| F002.9 | At the next sampling time, take【A to B assay plate】from the CO_2_ incubator to the clean bench, and then, move the BBB model insert to the next well of the assay plate. |  |
| F002.10 | After all the BBB model insert inserts and blank inserts have been moved, place back them on a shaker in a CO_2_ incubator (37 ℃, 5% CO_2_). | ・Repeat F002.9-10 at every sampling time. |
| F002.11 | After sampling, seal the top of the plate to prevent drying and store at -80 ℃ until measurement. |  |
| F002.12 | To perform a permeability assay from the blood vessel side to the brain side (B to A), prepare for 24 well plate**【**B to A assay plate**】**,the 96 well plate for the sample storage.  Add 900µl/well substrate solution in the 24 well plate and warm to 37 ℃.  Prepare a 24-well plate**【**Wash plate】for washing the culture insert inoculated with cells before the test.  Add 900µl/well D-PBSH and warm to 37 ℃. | ・Substrate solution should be vortexed well (more than 30 seconds)　before addition |
| F002.13 | **【B to A assay】**  Take the BBB model inserts from the CO_2_ incubator (33 ℃, 5% CO_2_) to the clean bench.  Wash the BBB model inserts to the【wash plate】before the assay. |  |
| F002.14 | Lift the BBB model inserts with tweezers, remove the medium from the insert by suction, and then return model to 【Wash plate】. | ・Performed for each insert |
| F002.15 | Add 200 µl of D-PBSH into the BBB model inserts and transfer from【Wash plate】to【B to A assay plate】.  Start counting up the timer and record the time on a recording sheet. |  |
| F002.16 | Repeat for the remaining BBB model inserts and blank inserts. Record the time on a recording sheet. |  |
| F002.17 | Place【B to A assay plate】on a shaker in a CO_2_ incubator (37℃, 5% CO_2_) (50 rpm) |  |
| F002.18 | When the sampling time is reached, remove the assay plate from the CO_2_ incubator to the clean bench, and then, transfer the 100µL of the buffer from the model to the 96well plate. | ・Do not pipette before sampling for avoiding the cell detachment. |
| F002.19 | Add 100µL of D-PBSH into the BBB model inserts |  |
| F002.20 | After all BBB model inserts and blank inserts have been collecting sample and adding the D-PBSH, place back them on a shaker in a CO_2_ incubator (37 ℃, 5% CO_2_). | ・Repeat F002.18～20 for the remaining BBB model inserts and blank inserts. |
| F002.21 | After sampling, pipette well the rest of the【A to B】and【B to A】substrate solution, and then, collect 100 µl each of the rest substrate solution on a 96 well plate.  Seal the top of the plate to prevent drying and store at -80 ℃ until measurement. |  |
| F002.22 | Quantify the substrate concentration to each substance and calculate the permeability coefficient. |  |
| - MEMO | | |

※No: Inside the Clean bench


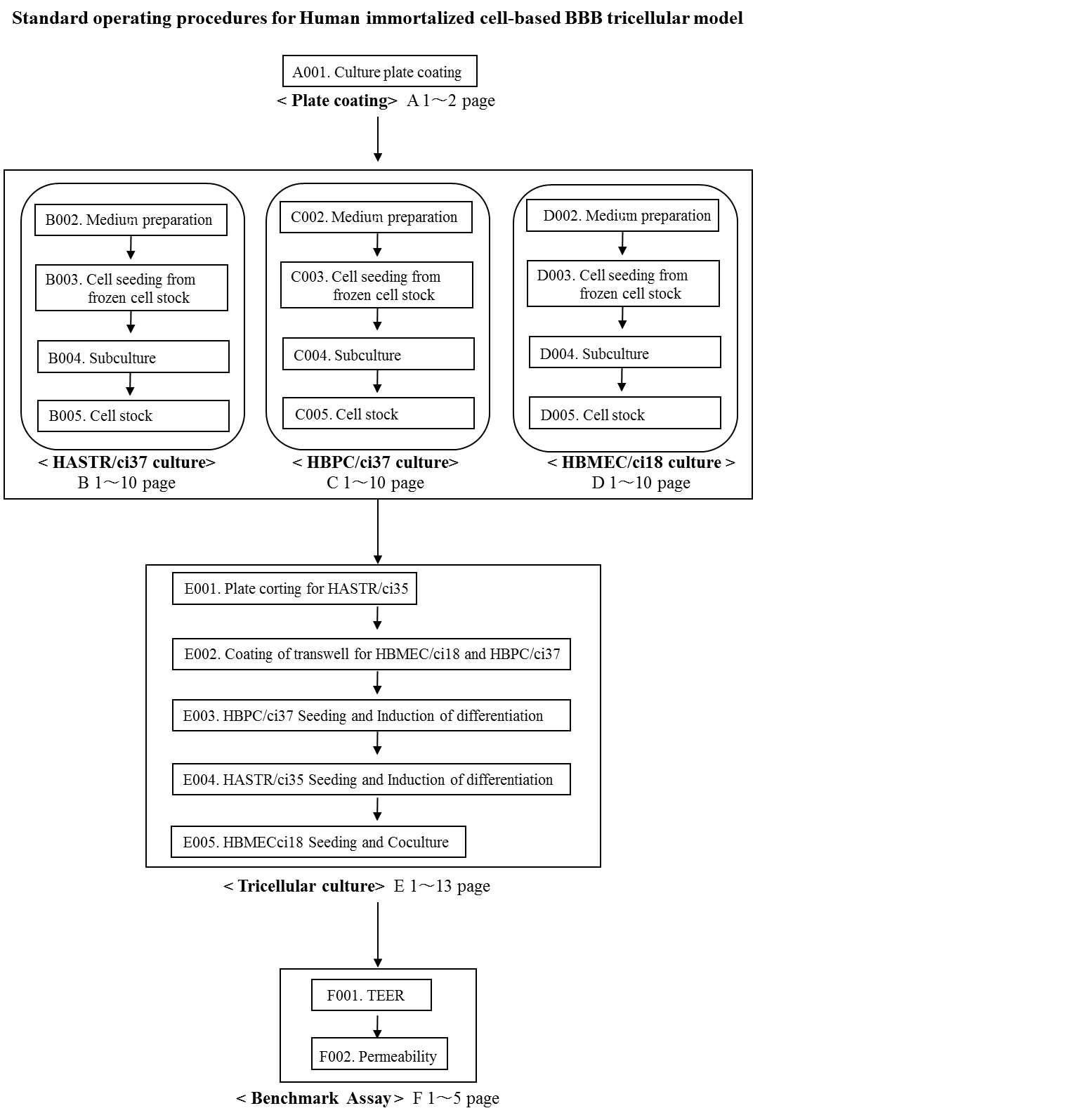

Supplement: Multimedia component 2 [file mmc2.docx]
